# Not Just a Matter of Space: Integrating Ecological Niche Modeling With Genotype‐Environment Associations Suggests High Maladaptation Risks Under Climate Change for a Microendemic Malagasy Frog

**DOI:** 10.1002/ece3.73664

**Published:** 2026-05-24

**Authors:** Francesco Belluardo, Mirko Di Febbraro, Javier Lobón‐Rovira, Ivo Oliveira Alves, Malalatiana Rasoazanany, Franco Andreone, Gonçalo M. Rosa, Simone Giovacchini, Enrico Mirone, Pushpinder Singh Jamwal, Sandra Afonso, Michele Innangi, Gabriella Sferra, Emiliano Trucchi, Alessandro Mondanaro, Francesco Carotenuto, Anna Loy, Angelica Crottini

**Affiliations:** ^1^ EnviXLab, Department of Biosciences and Territory University of Molise Pesche Italy; ^2^ CIBIO, Centro de Investigação em Biodiversidade e Recursos Genéticos, InBIO Laboratório Associado Universidade do Porto Vairão Portugal; ^3^ BIOPOLIS Program in Genomics, Biodiversity and Land Planning, CIBIO Vairão Portugal; ^4^ Museo Nacional de Ciencias Naturales (MNCN‐CSIC) Madrid Spain; ^5^ Departamento de Biologia Animal, Faculdade de Ciências Universidade de Lisboa Campo Grande Portugal; ^6^ Mention Zoologie et Biodiversité Animale, Domaine Sciences et Technologies Université d'Antananarivo Antananarivo Madagascar; ^7^ Museo Regionale di Scienze Naturali Torino Italy; ^8^ Biodiversity Research Institute (IMIB), CSIC‐Universidad de Oviedo‐Principality of Asturias Mieres Spain; ^9^ Institute of Zoology, Zoological Society of London London UK; ^10^ Centre for Ecology, Evolution and Environmental Changes (CE3C), Faculdade de Ciências, Universidade de Lisboa Lisboa Portugal; ^11^ Urban Biodiversity Laboratory, Department of Biosciences and Territory University of Molise Pesche Italy; ^12^ Department of Life and Environmental Sciences Marche Polytechnic University Ancona Italy; ^13^ Department of Earth, Environment and Resource Sciences University of Naples Federico II Napoli Italy; ^14^ Department of Biology University of Florence Sesto Fiorentino Italy

**Keywords:** ddRAD, genetic offset, habitat suitability, local adaptation, Madagascar, protected areas

## Abstract

Climate change is impacting biodiversity worldwide at an accelerating pace. Traditionally, Ecological Niche Models (ENMs) have been widely used to infer the likely impacts of climate change on species, while accounting for their potential dispersal towards climatically suitable habitats. More recently, Genotype‐environment association (GEA) approaches applied to genomic data have opened the possibility to investigate local adaptation underlying species' genetic adaptive capacity, allowing quantification of maladaptation risk under future climatic conditions. In this study, we integrate ENMs with GEAs to assess climate change impacts on *Mantidactylus bourgati* , a frog microendemic to the Andringitra Massif in southeastern Madagascar. ENMs forecasted a progressive decline in climatically suitable habitat that, depending on the climate change scenario, could lead to either complete extinction or a strong reduction and disjunct future distribution. GEA analyses suggested spatially structured genotype‐environment associations consistent with local adaptation, with three distinct adaptive units associated with the wide environmental gradients characterizing the Andringitra Massif region. Genetic offset calculations suggested that even if *M. bourgati* may succeed in tracking suitable habitats through dispersal, the future genetic change required to maintain the same adaptation to current climatic conditions will be significant, implying a high risk of maladaptation. Moreover, most future refugial habitats are projected to fall outside Madagascar's network of protected areas. These findings emphasize the importance of integrating species' genetic adaptive capacity into conservation strategies and spatial planning to help mitigate future climate change impacts on biodiversity.

## Graphical abstract

We integrate ecological niche modeling and genotype‐environment association analyses to jointly assess species responses to climate change in terms of dispersal potential and genetic adaptive capacity, and to estimate maladaptation risk under future scenarios in a microendemic Malagasy frog ( *Mantidactylus bourgati* ).

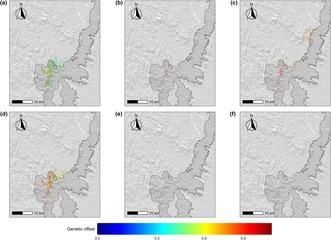

## 1 Introduction

Climate change is recognized as a major driver of the ongoing global biodiversity crisis (Bellard et al. 2012; Jaureguiberry et al. 2022; Luedtke et al. 2023; Pearson et al. 2014). Climate change effects are rapidly intensifying and are expected to be exacerbated by synergistic interactions with other anthropogenic threats (Di Febbraro et al. 2019; IPCC 2022; Mondanaro et al. 2024). Populations can respond to climate change by dispersing into new areas to track the change in the distribution of suitable habitats or persisting through genetic adaptation to local conditions (Bellard et al. 2012; Chen et al. 2011; Forester et al. 2025; Hoffmann and Sgrò 2011; Parmesan 2006; Richardson et al. 2014). Predicting and quantifying the effects of climate change on biodiversity is essential for effective species conservation.

Ecological Niche Models (ENMs) have become a widely adopted approach for investigating potential climate change impacts on biodiversity (e.g., Guisan and Thuiller 2005; López‐Tirado and Gonzalez‐Andújar 2023), supported by the growing availability of environmental datasets and species occurrence records in public repositories (e.g., GBIF.org 2025; Hijmans et al. 2005). ENMs characterize species' ecological requirements from occurrence data. These are then projected onto geographic landscapes and through time to generate maps of habitat suitability under specified climatic scenarios (Elith and Leathwick 2009; Miller 2010). While ENMs can characterize species' response to climate change as dispersal to climatically suitable habitats, they do not incorporate key aspects related to species' resilience—such as their genetic adaptive potential—therefore capturing only partially the complexity of species' responses to climate change (Hällfors et al. 2016; Razgour et al. 2019).

More recently, the integration of species' genetic adaptive capacity into studies predicting the nature and magnitude of climate change impacts on populations and species is constantly increasing (Capblancq et al. 2020; Razgour et al. 2019; Waldvogel et al. 2020). Populations can exhibit local adaptation, showing substantial variation in genetic composition and optimal fitness across geographic landscapes as a result of evolutionary responses to the heterogeneous distribution of environmental conditions (Capblancq et al. 2020; Hoste et al. 2024). Genomic signatures of local adaptation can be identified through Genotype‐Environment Association (GEA) approaches, which test the association between genomic variants and spatially explicit environmental variables measured at the sampling locations of genotyped individuals (Capblancq et al. 2018, 2020; Forester et al. 2018; Frichot et al. 2013). Modeling the GEAs allows us to quantify the genetic change required for populations to maintain their current local adaptation under future environmental conditions (Capblancq and Forester 2021; Fitzpatrick and Keller 2015). This measure, known as “genetic offset,” serves as a proxy for maladaptation risk (i.e., the degree to which species fail to adapt effectively to changing environmental conditions, leading to decreased fitness and increased extinction risk) and provides fundamental insights for species conservation (Brady et al. 2019; Capblancq et al. 2020; Rehfeldt et al. 2002).

Genetic offset can be spatially mapped, providing insights into where the species will face more difficulties in adapting to future climatic conditions (Capblancq and Forester 2021; Fitzpatrick and Keller 2015). Usually, these projections are generated using the whole current species' distributional range (e.g., Ferrer Obiol et al. 2025; Hoste et al. 2024). This approach could have limited value for practical conservation actions, since efforts to mitigate climate change effects could focus on areas where suitable habitat will no longer exist (due to range contraction) or fail to consider areas where the species could establish under future conditions (range expansion). In this context, integrating ENMs with genomic estimates of maladaptation risk by mapping genetic offset within areas of both current and future habitat suitability can provide more reliable predictions of species' maladaptation risk patterns and stronger guidance for effective conservation strategies.

Madagascar is one of the most celebrated biodiversity hotspots in the world, renowned for its unique combination of exceptionally high species richness and extreme levels of endemism (Antonelli et al. 2022; Myers et al. 2000). Amphibians are among the faunal groups whose native species are entirely endemic to the island and display astonishing levels of species richness, with over 430 described species (AmphibiaWeb 2025). Around 40% of Malagasy amphibians are microendemic, that is, characterized by extremely restricted distributional ranges (Brown et al. 2014, 2016), and several hypotheses have been proposed to explain this high frequency. Some emphasize the influence of evolutionary mechanisms, such as repeated population isolations in forested habitats during aridification periods associated with Quaternary climatic oscillations, occurring in lowland river catchments or within montane refugia (e.g., Brown et al. 2014, 2016; Raxworthy and Nussbaum 1995; Vences et al. 2009; Wilmé et al. 2006; Wollenberg et al. 2008, 2011). Others suggest that human activities might have contributed to range contractions in some species (Helmstetter et al. 2021; Orkin et al. 2025). Alternatively, narrow distributions of certain taxa may simply reflect insufficient field sampling (Antonelli et al. 2022; Carné, Vieites, Ferrer, et al. 2025).

With 47% of Malagasy amphibians classified in the highest threatened categories within the IUCN Red List (IUCN 2025), habitat loss and degradation are the primary threats, followed by overcollection for the pet trade, emerging infectious diseases, invasive species, and climate change (Andreone et al. 2021; Goodman 2022; Ralimanana et al. 2022). In particular, Madagascar is expected to experience rapid changes in climatic conditions, with projections indicating the loss of up to 50% of its biome surface under the most pessimistic climate change scenarios (Hannah et al. 2008; Lai and Beierkuhnlein 2024; Malcolm et al. 2006; Tadross et al. 2008). Early investigations suggested that montane frogs face the greatest risk from future shifts in climatically suitable habitats (Raxworthy et al. 2008). Recent studies have predicted substantial reductions, or even entire loss, of amphibians' potential distributions, requiring populations to undergo sharp range shifts to track suitable climatic conditions (Carné, Vieites, and Sillero 2025; Dubos et al. 2022, 2025; Edwards et al. 2022). Microendemic species are particularly vulnerable due to their limited distributions and expected narrow climatic niches, which limit their ability to reach future suitable areas that may lie far from their current ranges (Dubos et al. 2022; Edwards et al. 2022; Raxworthy et al. 2008). This is further aggravated by the potential shift of suitable areas outside the current network of Protected Areas (PAs), which could further hinder species' ability to disperse and track their climatic niches in space (Coldrey and Turpie 2021; Cui et al. 2025; Thomas and Gillingham 2015).

In this study, we predict the impact of climate change on *Mantidactylus bourgati* Guibé 1974, a small amphibian microendemic to the Andringitra Massif region in southeastern Madagascar, with an estimated Extent of Occurrence of 1313 km^2^ and listed as Endangered in the IUCN Red List (Belluardo et al. 2021; Guibé 1974; IUCN SSC Amphibian Specialist Group 2017). We explicitly integrated ENMs and GEA analyses to evaluate the species' response to current and future climate change conditions in terms of potential dispersal to suitable habitats and genetic adaptation to local conditions. Although *M. bourgati* occupies a broad elevation gradient within a mountainous landscape (930 to 2500 m a.s.l.; Belluardo et al. 2021; Guibé 1974), its geographic range is highly restricted and dispersal opportunities are presumably limited, rendering it particularly vulnerable to rapid climatic change and making it an ideal model for testing our methodological framework. Specifically, this study addresses the following objectives:

1. Assess the current and projected future distributions of *M. bourgati* under different climate change scenarios as predicted through ENMs.
2. Investigate genomic signatures indicating local adaptation of *M. bourgati* populations to current climatic conditions.
3. Quantify the species' adaptive capacity by identifying genetic adaptive units under current climatic conditions and forecasting their geographic distribution under future climate change scenarios.
4. Quantify the risk of maladaptation in terms of genetic offset and characterize its geographic distribution across projected future suitable habitats.
5. Determine the extent of geographic overlap among habitat suitability, adaptive units, and genetic offset values with Madagascar's existing PA network under current and future climate scenarios.

## 2 Materials and Methods

### 2.1 Study Area and Species

**FIGURE 1:**
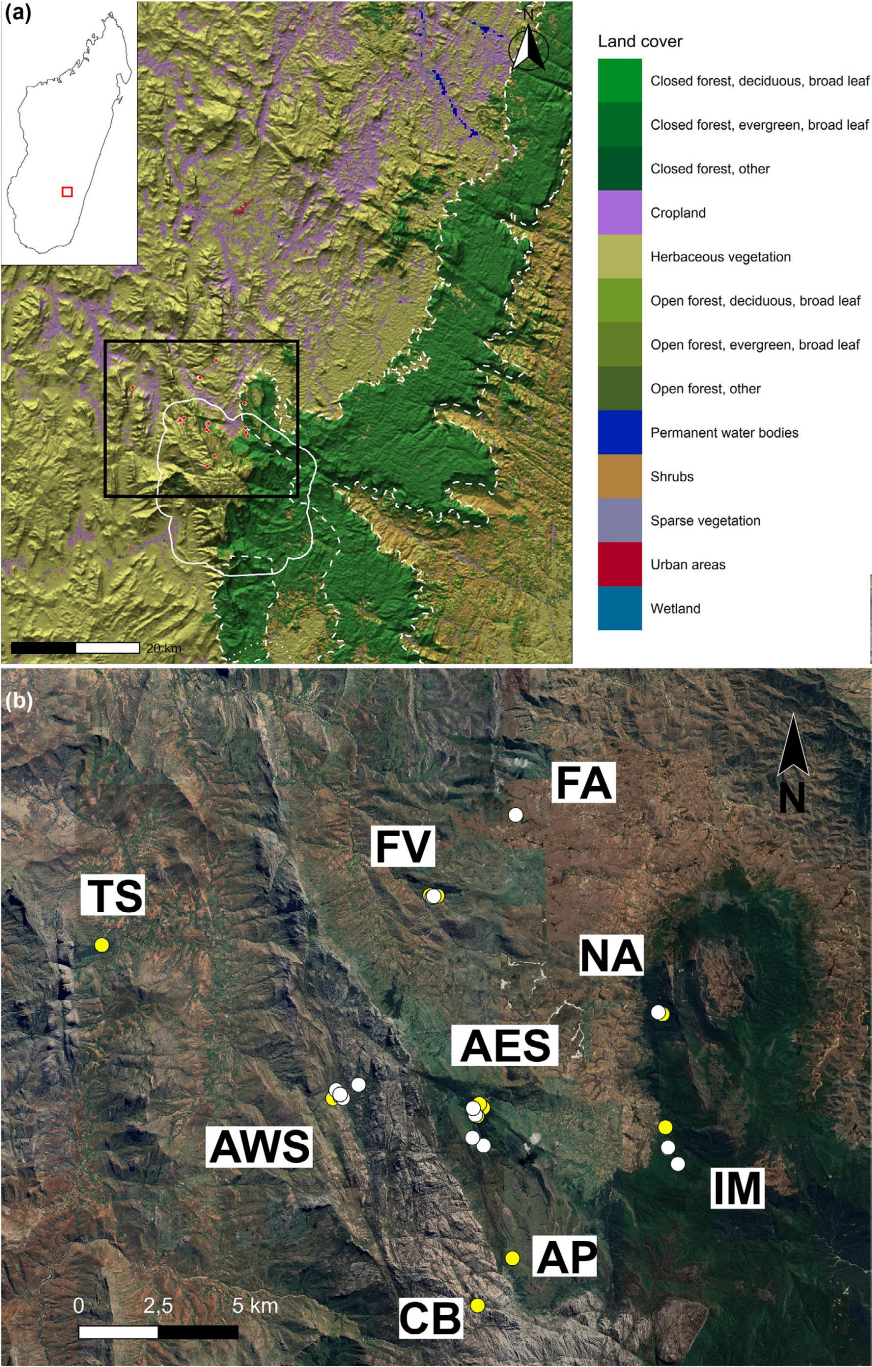
Study area. (a) Land cover map of the Andringitra region (derived from European Union's Copernicus Land Monitoring Service; https://doi.org/10.2909/c6377c6e‐76cc‐4d03‐8330‐628a03693042); red dots indicate species' occurrences used for Ecological Niche Models; protected area boundaries are indicated by white outlines: Solid line—Andringitra National Park; dashed line—Paysage Harmonieux Protégé du Corridor Forestier Ambositra–Vondrozo; dotted line—Pic d'Ivohibe Special Reserve; the black rectangle delineates the extent of the map shown in panel (b). (b) Distribution of geographic occurrence records (yellow) and genetic samples (white; included in Genotype‐environment association analyses); population/locality codes: AES, Andringitra Eastern Slopes; AP, Andohariana Plateau; AWS, Andringitra Western Slopes; CB, Cuvette Boby; FA, Fivahona Ambavanala; FV, Fivahona Velotsoa; IM, Imaitso; NA, Namoly; TS, Tsaranoro; map imagery 2024 Google. See Table S1 for localities coordinates.

The Andringitra Massif, located in southeastern Madagascar, lies between the eastern escarpment and the central high plateau (Figure 1) and includes the country's second‐highest peak (Pic Boby; 2658 m a.s.l.) (Goodman 1996; Nicoll and Langrand 1989). It spans two distinct bioclimatic regions, with the eastern slopes falling within the humid rainforest biome and the western slopes within the sub‐humid forest biome (Goodman 1996). This region hosts a large diversity of habitats, including lowland rainforest, semi‐arid deciduous forest, montane meadows, heathlands, and rocky outcrops (Belluardo et al. 2021; Goodman 1996; Goodman et al. 2018). Several PAs encompass this region: the Andringitra National Park, covering the core of the Massif; the Paysage Harmonieux Protégé du Corridor Forestier Ambositra–Vondrozo, protecting the Ambositra–Vondrozo forest corridor; and the Pic d'Ivohibe Special Reserve (Figure 1; Goodman et al. 2018).

*Mantidactylus bourgati* is a small frog (maximum snout‐vent length 40 mm; Glaw and Vences 2007) microendemic to the Andringitra Massif region (Belluardo et al. 2021; Guibé 1974). The species appears to be primarily associated with clean‐water streams, where it breeds by larval development, occurring both in forested habitats and in montane grasslands above the tree line (Belluardo et al. 2021; Glaw and Vences 2007; IUCN SSC Amphibian Specialist Group 2017). Main threats to the species' conservation include habitat loss driven by overgrazing and fires (Belluardo et al. 2021; IUCN SSC Amphibian Specialist Group 2017).

### 2.2 Tissue Sampling and Occurrence Dataset Compilation

Samples analyzed in this study were collected in 2018 during a herpetological survey conducted in the Andringitra Massif region (Belluardo et al. 2021). We collected tissue samples from 40 individuals of *M. bourgati* across six localities encompassing the western and eastern sides of the Massif, recording the geographic coordinates of collection sites. Sampling localities encompass the overall areas where the species is confidently known to occur (Figure 1; Table S1; see Belluardo et al. 2021 for details on sampling activities). In addition, we enriched the *M. bourgati* dataset including additional geographic records from the same survey and published sources (Brown et al. 2014). Only records with precise and verifiable localities were retained. In addition, we applied a rarefaction procedure to assure that no more than a single point fell within a 100 × 100 m raster cell used as resolution for the environmental variables (see below; Di Febbraro et al. 2017), resulting in a final pool of 28 occurrences used in ENMs calibration. Sampling procedures complied with international, national, and institutional guidelines for amphibian tissue sampling, and fieldwork was conducted under permits issued by the relevant Malagasy authorities.

### 2.3 Ecological Niche Modeling

#### 2.3.1 Environmental Variables

To calibrate ENMs, we considered the 19 bioclimatic variables provided in the CHELSA database (ver. 2.1; Karger et al. 2023), originally rasterized at 1 km spatial resolution. After cropping the 19 variables over the study area, they underwent a downscaling procedure to a resolution of 100 × 100 m (Text S1; Table S2). We also considered the 100 × 100 m resolution digital elevation model (DEM) provided by Jarvis et al. (2008), which was used to derive four additional topographic indices (i.e., slope, aspect, roughness and water flow direction; Wilson et al. 2007). After checking for multicollinearity using a threshold of Pearson's |*r*| ≤ 0.75, the final predictor set was reduced to nine (isothermality, temperature seasonality, temperature annual range, mean temperature of driest quarter, precipitation of wettest month, precipitation of driest month, aspect, flow direction, slope; Table S3). These analyses were carried out using the “terra” and “usdm” R packages (Hijmans 2025; Naimi et al. 2014).

#### 2.3.2 Model Calibration

ENMs were calibrated adopting the so‐called “ensemble of small models” framework, an approach specifically designed to prevent model overfitting issues (Breiner et al. 2015) that can arise when there are few occurrences compared to the number of environmental variables (Guisan and Zimmermann 2000). Accordingly, we calibrated a set of bivariate models, that is, considering all possible combinations of predictor pairs and then averaging the results of each model. Bivariate models were trained by using five algorithms: Generalized Linear Model (GLM), Generalized Additive Model (GAM), Generalized Boosting Model (GBM), Random Forest (RF) and Maximum Entropy (MAXENT). To define the “M” area (i.e., the region that has been accessible to the species via dispersal over relevant periods of time) under the framework by Barve et al. (2011), we derived the major watersheds in the study area from the digital elevation model map (Brenning et al. 2025) and used the resulting region as the domain where to place 10,000 background points for ENMs calibration (Karami et al. 2024; Lo Parrino et al. 2023; Nori and Rojas‐Soto 2019). Moreover, to account for potential sampling biases in species occurrences, background points were geographically placed according to the density of the occurrence data, so that more background points fell where presences are denser (Di Febbraro et al. 2023; Mondanaro et al. 2021; Roy‐Dufresne et al. 2019). The predictive performance of ENMs was assessed using a block cross‐validation approach (Muscarella et al. 2014), that is, splitting data into four geographically nonoverlapping bins of equal occurrence number, corresponding to each corner of the entire geographical space. This method has been used to assess model transferability, that is, the ability to extrapolate predictions into new areas (Muscarella et al. 2014), as well as to penalize models based on biologically meaningless predictors (Fourcade et al. 2018). The predictive performance of each model was assessed by measuring the area under the receiver operating characteristic curve (AUC; Hanley and McNeil 1982) and the Continuous Boyce Index (CBI; Hirzel et al. 2006). In addition, we dropped poorly calibrated models (i.e., achieving AUC < 0.7; Di Febbraro et al. 2023) from the subsequent analyses. Ensemble models were obtained by averaging the projections of individual models, weighted by their respective AUC scores (Marmion et al. 2009). ENMs were projected on current climate and on two future climate change scenarios from the Coupled Model Intercomparison Project Phase 6 (CMIP6; O'Neill et al. 2016): SSP1‐2.6, which predicts global warming to remain < 2°C compared to preindustrial level; and SSP5‐8.5, which forecasts a temperature increase of 2.40°C–5.57°C by 2100 (Tebaldi et al. 2021). For both future scenarios, ENMs spatial predictions considered four‐time intervals according to the climate projections available in the CHELSA database, that is, 1981–2010 as current time (labeled as “2010”, hereafter), 2011–2040 (“2040”), 2041–2070 (“2070”), and 2071–2100 (“2100”). Since different global circulation models may lead ENMs to predict diverging climate change effects (Buisson et al. 2010), we considered four alternative versions, as generated by the GFDL–ESM4, IPSL–CM6A–LR, MPI–ESM1–2–HR, and UKESM1–0–LL global circulation models available in the CHELSA database (see also Sanderson et al. 2015). ENMs were generated using the “biomod2” (ver. 4.2.3) and “ecospat” (ver. 4.1) R packages (Broennimann et al. 2025; Thuiller et al. 2025). To assess the effect of model extrapolation on climate predictor values falling outside the calibration range, we calculated multivariate environmental similarity surfaces (i.e., MESS; Elith et al. 2011). Current and future ENMs projections were binarized to obtain range maps according to three threshold approaches (i.e., “maximize TSS”, “minimum observed presence”, and “10th percentile” Liu et al. 2013), to account for the effect of using different binarization schemes (Di Febbraro et al. 2018). Since thresholding continuous suitability values can determine under/overprediction issues (D'Amen et al. 2015) in ENM projections and, more generally, a loss of information on gradients in species‐habitat relationships, we created additional versions of the binary maps by considering a 200 m radius buffer around binary suitable patches and replicating all the subsequent analyses with these alternative maps. Lastly, future ENM projections were spatially constrained accounting for the species' ability to disperse in future favorable environments. For this purpose, we used the “MigClim” R package (Engler et al. 2012), a cellular automaton model that adjusts future projections accounting for species‐specific dispersal limitations. As no exhaustive data are available on the average dispersal distance of the species, we relied on the evidence provided by Andreone et al. (2013) and implemented three alternative dispersal scenarios in MigClim, that is, 50, 100, and 200 m/year.

### 2.4 Genomic Data

**FIGURE A1:**
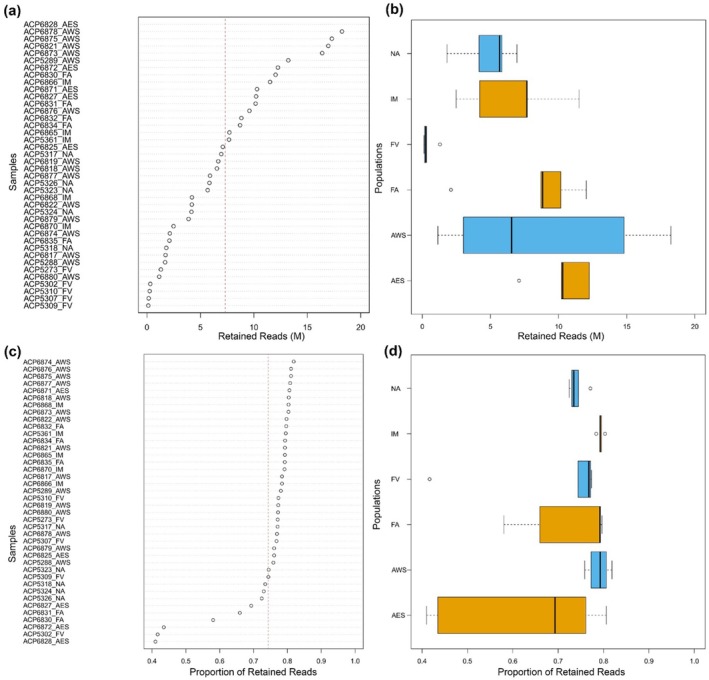
Distribution of retained reads following “process_radtags” filtering (demultiplexing, quality filtering, and adapter trimming). (a) Number of retained reads (in millions) per sample, sorted in ascending order. (b) Distribution of retained reads per population. (c) Proportion of retained reads relative to total input reads per sample, sorted in ascending order. (d) Distribution of proportion of retained reads relative to total input reads per population. Red dashed lines in a and c indicate mean values across all samples. In box plots, boxes represent the interquartile range (IQR), with the median shown as a vertical line; whiskers extend to 1.5 × IQR, and points beyond represent outliers. Samples from Fivahona Velotsoa were excluded from subsequent analyses due to low retained read counts (see Text S2 for details). Populations codes: AES, Andringitra Eastern Slopes; AWS, Andringitra Western Slopes; FA, Fivahona Ambavanala; FV, Fivahona Velotsoa; IM, Imaitso; NA, Namoly. See Figure 1 for population distribution across the study area. Plots were generated with R scripts from Rivera‐Colón and Catchen (2022).

**FIGURE A2:**
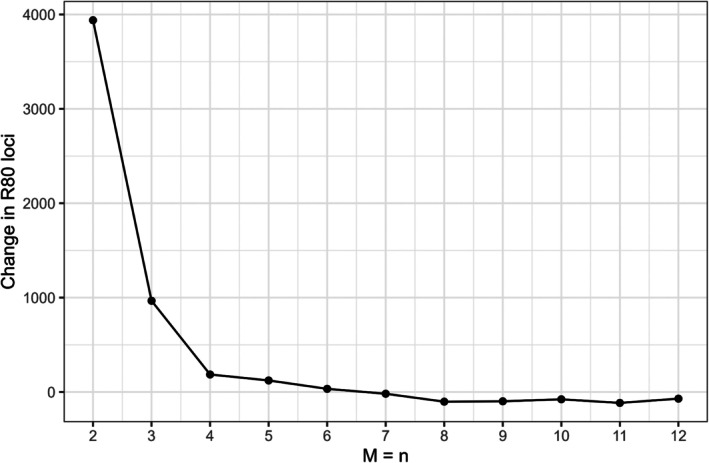
Optimization of de novo assembly parameters. Change in number of polymorphic loci present in at least 80% of the samples (R80) across subsequent iterations of the “denovo_map.pl” pipeline, with parameters ‐M and ‐n set equal to each other and iteratively tested for values ranging from 1 to 12 on a subset of 12 samples. The selected optimal values (*M* = *n* = 6) correspond to the smallest yet positive increase in R80 loci between iterations.

**FIGURE A3:**
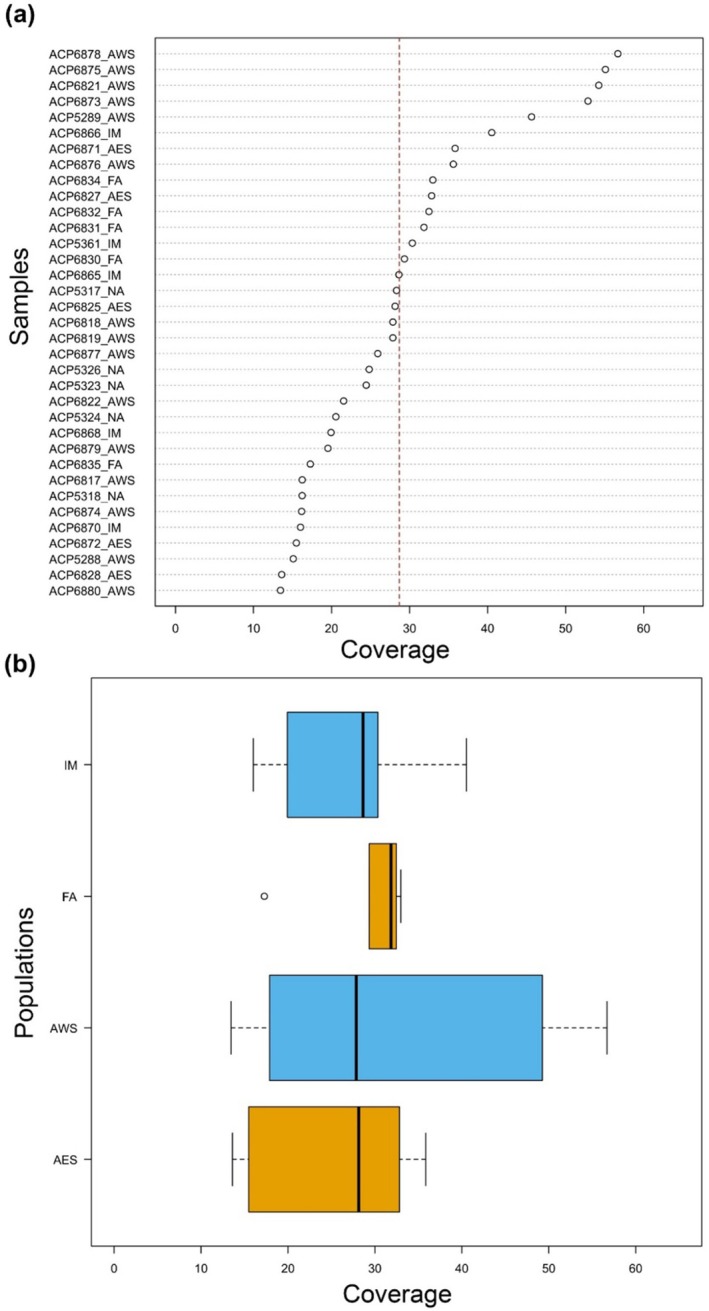
Distribution of coverage following the gstacks step of the de novo assembly on the full dataset. (a) Average coverage per sample calculated as the mean coverage across loci weighted by the number of individuals sharing each locus. (b) Distribution of average coverage per population. The vertical red dashed line in (a) indicates mean values across all samples. In box plots, boxes represent the interquartile range (IQR), with the median shown as a vertical line; whiskers extend to 1.5 × IQR, and points beyond represent outliers. Samples ACP5288, ACP6817, and ACP6880 from the Andringitra Western Slopes population were excluded from subsequent analyses due to low coverage levels (see Text S2 for details). Populations codes: AES, Andringitra Eastern Slopes; AWS, Andringitra Western Slopes; FA, Fivahona Ambavanala; IM, Imaitso; NA, Namoly.

The detailed procedure for genomic data generation, including library preparation and sequencing, and assembly is provided in Text S2, Table S4 and Figures A1, A2, A3. Briefly, we generated a Double‐digest Restriction‐Site Associated DNA (ddRAD) dataset and processed raw reads with Stacks 2.6 (Catchen et al. 2013) for demultiplexing, quality filtering, de novo assembly (given the absence of available reference genomes for the target species or closely related species), and SNP calling. SNP filtering was performed using the “populations” module, retaining only those genotyped in at least 85% of individuals, with a minor allele count of at least two, and a maximum observed heterozygosity of 0.5. To minimize linkage among SNPs, a single SNP per locus was randomly selected. After excluding all five samples from the Fivahona Velotsoa (FV) population and three samples from the Andringitra Western Slopes (AWS) population due to low numbers of retained reads and high proportions of missing genotypes (see Text S2 for details), the final dataset used in downstream analyses included 32 individuals and 6381 SNPs.

### 2.5 Population Genetic Structure and Patterns of Isolation‐By‐Distance

Population genetic structure (along with the subsequent GEA analyses; see below) was investigated using the Life on the Edge (LotE) pipeline, which integrates different steps of GEA analyses into a unified set of R functions (Barratt et al. 2024). We first explored population structure using Principal Component Analysis (PCA), computed with the “vegan” R package (Oksanen et al. 2025). Individual ancestry and admixture coefficients were estimated through sparse nonnegative matrix factorization (sNMF; Frichot et al. 2014), implemented in the “LEA” R package (Frichot and François 2015). We tested *K* ancestral population values ranging from 1 to 15, with 10 replicated runs per *K*. The optimal *K* value was determined using the cross‐entropy criterion, selecting the value associated with the lowest mean cross‐entropy across replicates. Finally, we tested for isolation‐by‐distance (IBD) through a Mantel test to assess the correlation between genetic distances (calculated as the inverse of the proportion of shared alleles) and geographic distances (computed as Euclidean distances between UTM coordinates) among individuals, using the “vegan” R package (Oksanen et al. 2025).

### 2.6 Genotype‐Environment Association Analyses

The following GEA analyses were performed with the LotE pipeline (Barratt et al. 2024). Given the limited spatial extent of the study area and the restricted sample size related to the microendemic status of the species, we adopted a conservative GEA framework explicitly accounting for population structure, integrating complementary GEA methods, and evaluating sensitivity of GEA tests for outlier detection (see below) to minimize confounding effects and reduce the risks of false‐positive associations.

**FIGURE A4:**
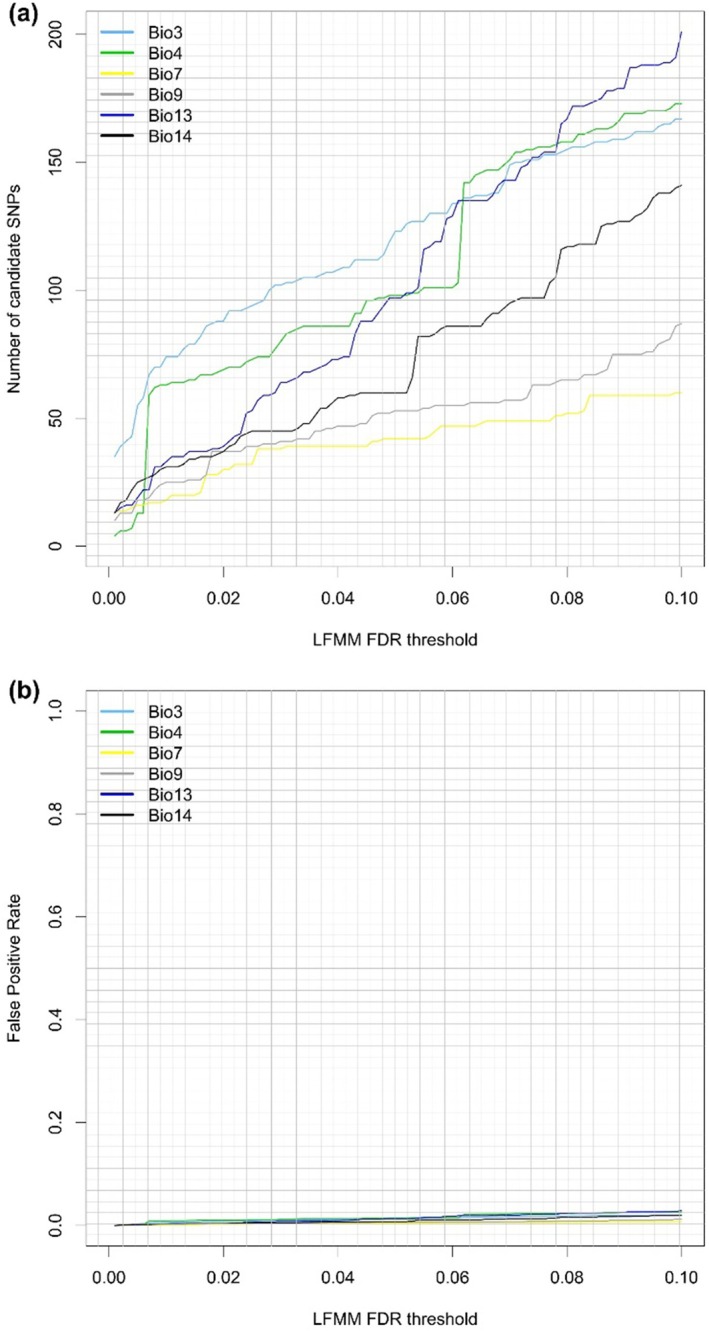
LFMM sensitivity analyses across varying False Discovery Rate (FDR) thresholds (from 0.001 to 0.1). (a) Number of candidate SNPs detected for each bioclimatic predictor across the range of tested FDR thresholds. (b) Estimated false positive rate of candidate SNPs across the range of tested FDR thresholds for each bioclimatic variable. Bioclimatic predictors description: Bio3—Isothermality; Bio4—Temperature seasonality; Bio7—Temperature annual range; Bio9—Mean temperature of driest quarter; Bio13—Precipitation of wettest month; Bio14—Precipitation of driest month.

**FIGURE A5:**
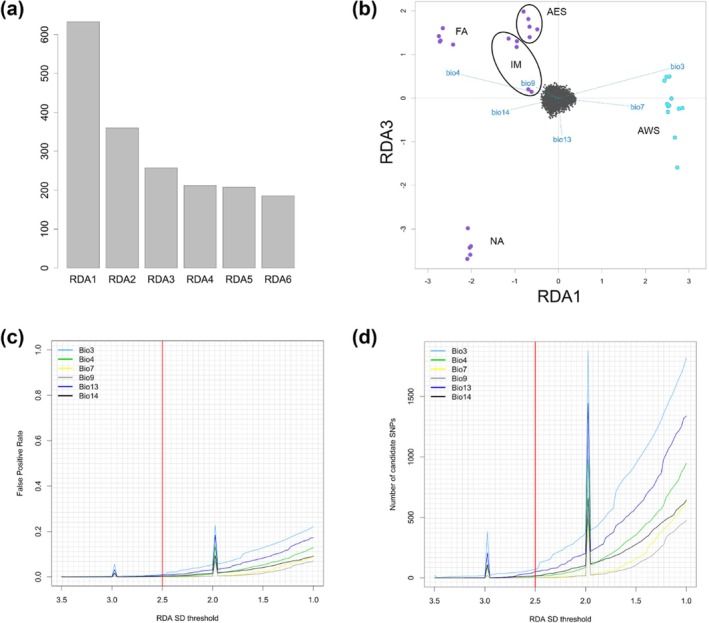
Identification of candidate SNPs under selection with RDA. (a) Screeplot showing the constrained inertia explained by each RDA axis. (b) RDA1 vs. RDA3 scatterplot with individuals colored based on the two genetic clusters identified with the sparse nonnegative matrix factorization (sNMF) method (see Figure 4b); gray dots in the middle represent SNPs; ellipses highlight individuals from the Andringitra Eastern Slopes and Imaitso populations. (c) RDA sensitivity analysis of the estimated false positive rate of candidate SNPs across a range of tested standard deviation (SD) thresholds (from 1.0 to 3.5) for each bioclimatic variable. (d) RDA sensitivity analysis of the number of selected candidate SNPs relative to a range of tested SD thresholds (from 1.0 to 3.5) for each bioclimatic variable. The vertical red lines indicate the SD threshold applied in the definitive analysis. See Figure 1 for population distributions. Populations codes: AES, Andringitra Eastern Slopes; AWS, Andringitra Western Slopes; FA, Fivahona Ambavanala; IM, Imaitso; NA, Namoly. Bioclimatic predictors description: Bio3, Isothermality; Bio4, Temperature seasonality; Bio7, Temperature annual range; Bio9, Mean temperature of driest quarter; Bio13, Precipitation of wettest month; Bio14, Precipitation of driest month.

Loci putatively under selection were identified using Latent Factor Mixed Models (LFMM; Frichot et al. 2013) and Redundancy Analysis (RDA; Capblancq et al. 2018; Forester et al. 2018). Loci were identified based on their association to the six climatic variables among the nine used for ENMs (Table S3). Missing SNP genotypes were imputed using two different approaches, depending on the analysis. For LFMM analyses, we applied the matrix completion approach implemented in the “LEA” R package (Frichot and François 2015). For RDA, missing values were imputed using mean genotype frequencies calculated within genetic clusters identified through sNMF analyses, following Razgour et al. (2018). LFMM analyses were performed with the “lfmm” R package (Caye et al. 2019). After optimizing LFMM parameters and exploring its sensitivity for outlier SNP detection (see Text S2; Figure A4), we executed the final LFMM analysis with 5 *K* latent factors and retained SNPs identified with a False Discovery Rate of 0.05. RDA was performed with the “vegan” R package (Oksanen et al. 2025). We retained RDA loadings of the first three axes based on their proportion of explained inertia (Figure A5a). SNPs with extreme loadings on these axes were considered putatively under selection. After exploring RDA sensitivity in SNP selection (see Text S2; Figure A5), we retained outlier SNPs based on a standard deviation (SD) threshold of 2.5 used as selection cutoff. For downstream analyses, we retained SNPs that were jointly detected by both LFMM and RDA.

### 2.7 Identification of Adaptive Units (AU)

The scores in the RDA space of the SNPs putatively under selection were grouped by implementing a specific clustering procedure (Text S3; Table S5) to identify distinct adaptive units (AUs) among *M. bourgati* individuals. To quantify the differential contribution of the six climatic variables in driving the separation among the AUs in the RDA space, we calibrated a random forest (RF; Breiman 2001) classification model setting the AUs determined by the clustering procedure as the response variable, and the values of the climatic variables extracted from the occurrence pixels of the SNPs under selection as the covariates. RF predictive performance was assessed through classification accuracy calculated under a 10‐fold cross‐validation scheme, while variable importance was quantified calculating the Gini index. Lastly, RF was projected into the geographic space, thus generating a spatial distribution of the AUs identified by the clustering procedure. AUs spatial projections were generated within the binary presence pixels as predicted by ENMs considering each combination of binarization threshold, time step, GCM, and climate change scenarios. RF was calibrated with the “caret” R package (Kuhn 2008).

### 2.8 Genetic Offset Calculation

**FIGURE A6:**
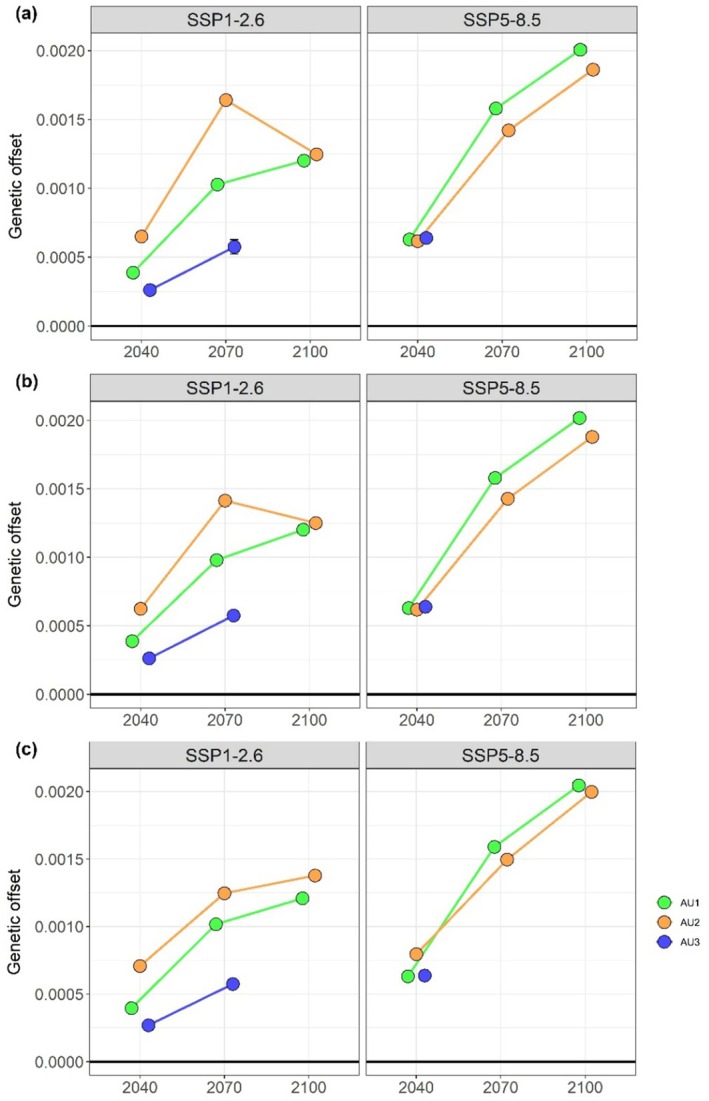
Genetic offset for *M. bourgati* estimated using the Gradient Forest (GF) approach under two climate change scenarios. Genetic offset values were projected in geographic space within the Ecological Niche Model‐predicted suitable areas of each adaptive unit (AU) across four‐time steps (2010–2100) under SSP1‐2.6 and SSP5‐8.5 scenarios. Dots indicate mean values calculated across multiple binarization thresholds and global circulation models, while whiskers refer to standard deviation values. (a) Genetic offset assuming a dispersal rate of 50 m/year. (b) Genetic offset assuming a dispersal rate of 100 m/year. (c) Genetic offset assuming a dispersal rate of 200 m/year.

**FIGURE A7:**
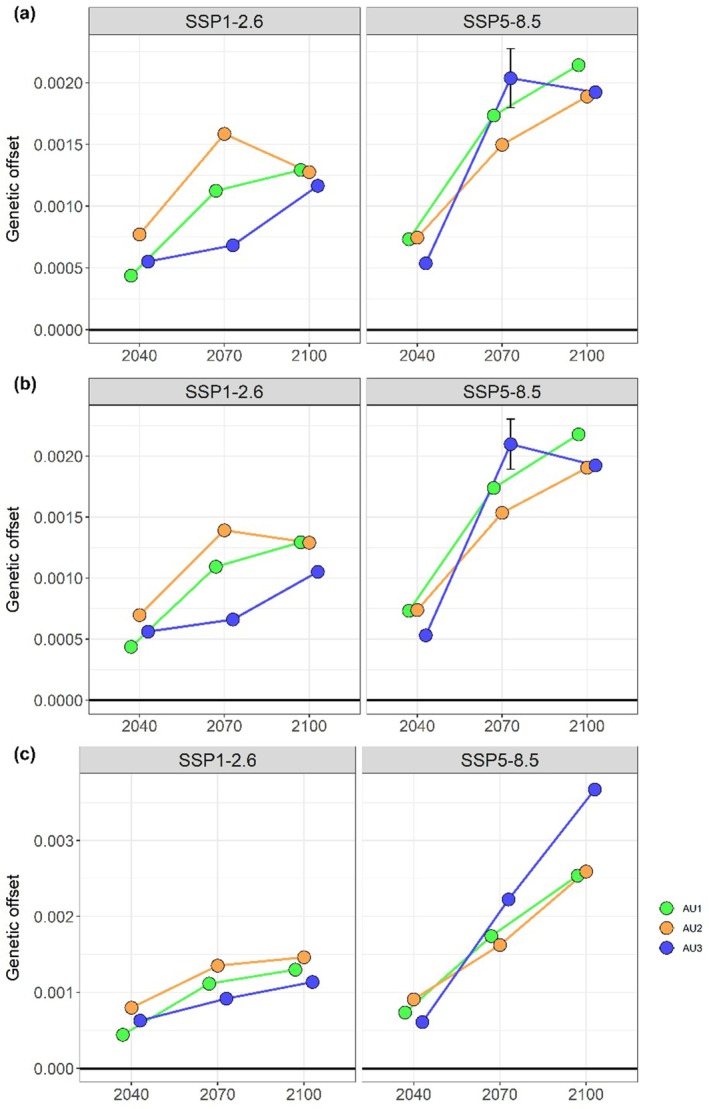
Genetic offset for *M. bourgati* estimated using the Gradient Forest (GF) approach under two climate change scenarios, with a 200 m radius buffer around binary suitable patches. Genetic offset values were projected in geographic space within the Ecological Niche Model‐predicted suitable areas of each adaptive unit (AU) across four‐time steps (2010–2100) under SSP1‐2.6 and SSP5‐8.5 scenarios. Dots indicate mean values calculated across multiple binarization thresholds and global circulation models, while whiskers refer to standard deviation values. (a) Genetic offset assuming a dispersal rate of 50 m/year. (b) Genetic offset assuming a dispersal rate of 100 m/year. (c) Genetic offset assuming a dispersal rate of 200 m/year.

We calculated genetic offset using the LotE pipeline (Barratt et al. 2024), based on RDA loadings derived from the set of SNPs putatively under selection. We retained the first three RDA axes to ensure consistency with the analyses performed on the full SNP dataset for candidate SNP identification. Specifically, we computed the global genetic offset, which integrates genetic offset values across all retained RDA axes. Genetic offset was calculated based on Euclidean distances calculated for each pixel between current and future weighted RDA scores, with future projections generated using the same combinations of time steps, GCMs, and climate change scenarios as considered in ENMs. Genetic offset values calculated for each of these combinations were then projected in the geographic space within the binary presence pixels as predicted by ENMs considering each binarization threshold. To additionally account for potential nonlinear genotype‐environment associations, we implemented a Gradient Forest (GF) approach for genetic offset calculation as a complementary analysis (see Text S2 for details; Figures A6 and A7).

### 2.9 Protected Area Coverage of Suitable Habitat, Genetic Offset, and Adaptive Units

To assess the effectiveness of existing PAs in covering *M. bourgati* populations under current and future suitable habitat, as well as AUs distribution and genetic offset values, we quantified the values of these three metrics inside and outside Madagascar's PA network, as provided in the World Database of Protected Areas (UNEP‐WCMC 2019).

## 3 Results

### 3.1 Ecological Niche Models

**FIGURE 2:**
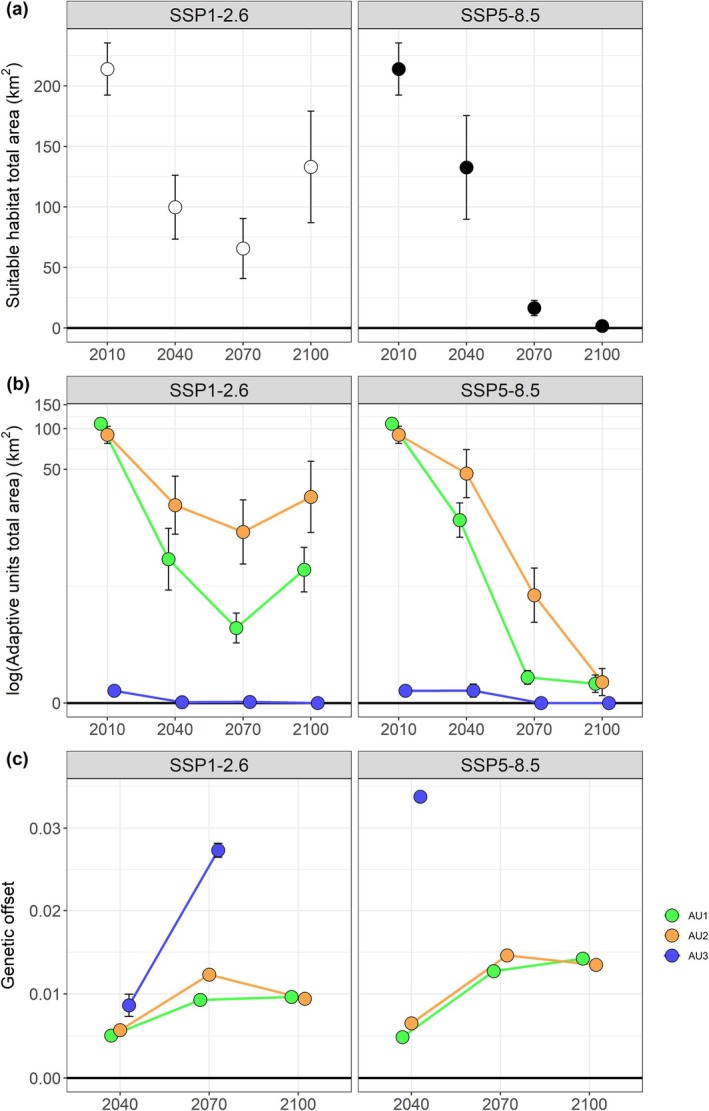
Extent of suitable habitat for *M. bourgati* as predicted by Ecological Niche Models (ENMs), spatial projections of adaptive units (AUs), and genetic offset values. Suitable areas were estimated across four‐time steps (2010–2100) under two climate change scenarios (SSP1‐2.6 and SSP5‐8.5), assuming a dispersal rate of 100 m/year. Dots indicate mean values calculated across multiple binarization thresholds and global circulation models, while whiskers refer to standard deviation values. (a) Total suitable area over time. (b) Total area of AUs spatial projections within the ENM‐predicted suitable areas (see Figure 4d for AU clustering details). (c) Genetic offset values projected in geographic space within the ENM‐predicted suitable areas of each AU.

**FIGURE A8:**
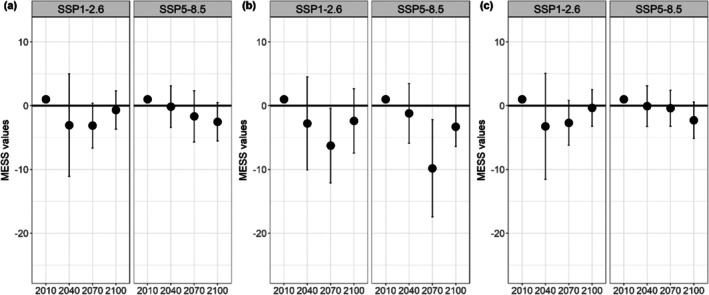
Multivariate environmental similarity surfaces (MESS) values computed for ENM of current and future projections under the three dispersal scenarios simulated using the ‘MigClim’ R package. Panels represent different annual dispersal distances: (a) 50 m/year, (b) 100 m/year; (c) 200 m/year.

ENMs showed a good predictive performance *sensu* Swets (1988), achieving a mean AUC value equal to 0.85 (SD = 0.21) and a mean CBI value of 0.74 (SD = 0.28). According to MESS results, ENMs reported a negligible extrapolation (i.e., MESS < −20; *sensu* Iannella et al. 2017; Figure A8) when predicting occurrence probability in future scenarios. By averaging the total suitable habitat area across the different binarization thresholds and global circulation models, projections suggested an overall decline in habitat suitability for *M. bourgati* from 2010 to 2100 (Figure 2a).

**FIGURE 3:**
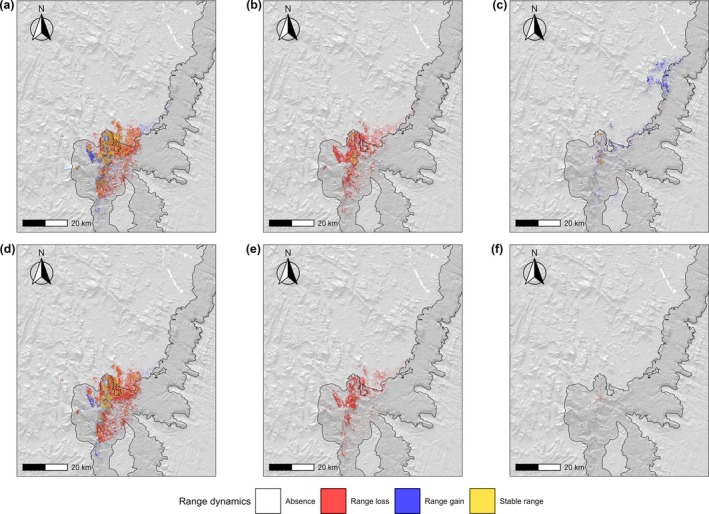
Temporal dynamics in habitat suitability for *M. bourgati* as predicted by Ecological Niche Models (ENMs) from 2010 to 2100 under two climate change scenarios. Panels (a–c) show projections under SSP1‐2.6, while panels (d–f) show projections under SSP5‐8.5. Time intervals correspond to: (a, d) 2010–2040; (b, e) 2040–2070; (c, f) 2070–2100. Projections depicted in the figure are based on the 100 m/year dispersal scenario and were generated using the maximize TSS binarization threshold and the IPSL–CM6A–LR global circulation model. Protected area boundaries are outlined in black and filled with a darker gray tone (see Figure 1 for details).

However, the magnitude of this decline varied substantially between the two examined climate change scenarios. Under SSP1‐2.6, the total suitable habitat area available in 2010 (approximately 220 km^2^) decreased steadily to about 70 km^2^ by 2070, before partially recovering by 2100 to nearly 140 km^2^. In contrast, under the most pessimistic scenario SSP5‐8.5, the declining trend persisted monotonically throughout the century, culminating in the complete loss of suitable habitat by 2100. Between 2010 and 2040, a large portion of the currently suitable area is either maintained or lost, in varying proportions depending on the scenario considered, with only slight range expansion particularly towards the west, south, and northeast (Figure 3a,d).

**FIGURE A9:**
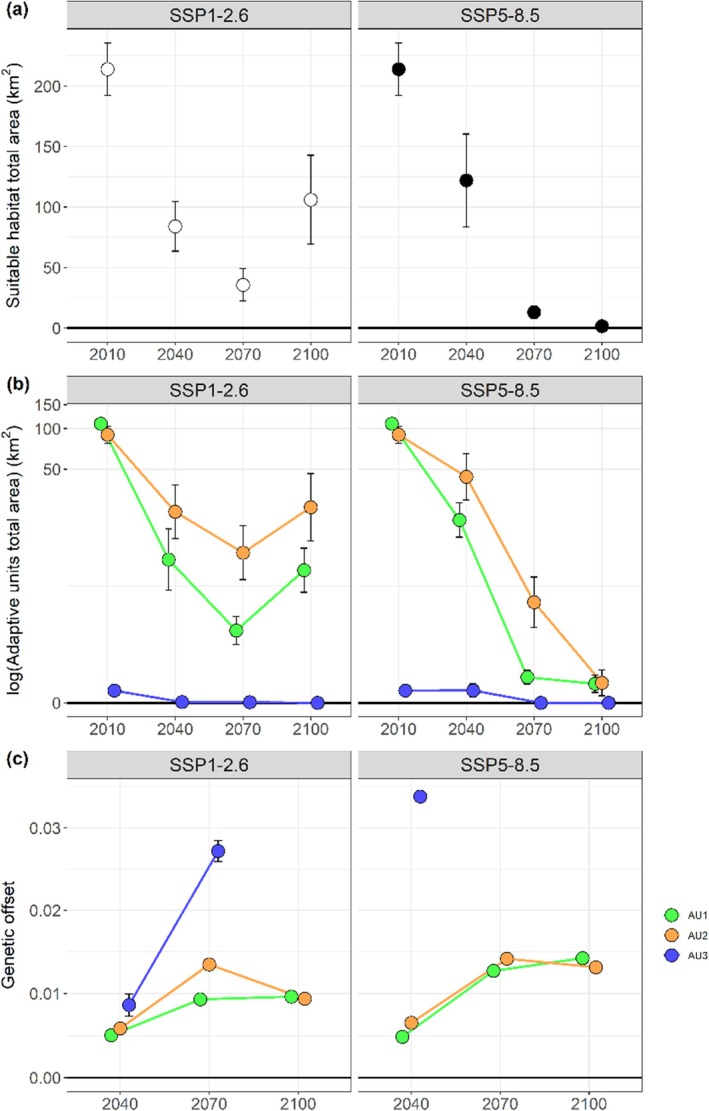
Extent of suitable habitat for *M. bourgati* as predicted by Ecological Niche Models (ENMs), spatial projections of adaptive units (AUs) and genetic offset values. Suitable areas were estimated across four‐time steps (2010–2100) under two climate change scenarios (SSP1‐2.6 and SSP5‐8.5), assuming a dispersal rate of 50 m/year. Dots indicate mean values calculated across multiple binarization thresholds and global circulation models, while whiskers refer to standard deviation values. (a) Total suitable area over time. (b) Total area of AUs spatial projections within the ENM‐predicted suitable areas (see Figure 4d for AU clustering details). Colors represent different AUs (AU1—yellow; AU2—blue; AU3—red). (c) Genetic offset values projected in geographic space within the ENM‐predicted suitable areas of each AU.

**FIGURE A10:**
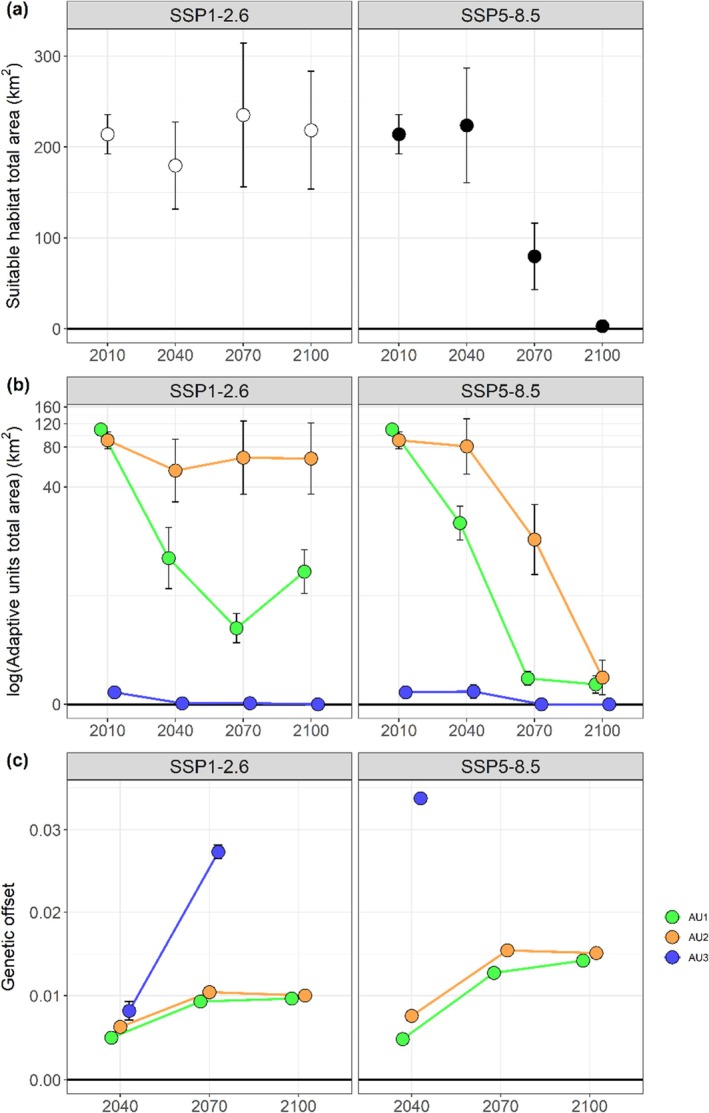
Extent of suitable habitat for *M. bourgati* as predicted by Ecological Niche Models (ENMs), spatial projections of adaptive units (AUs) and genetic offset values. Suitable areas were estimated across four‐time steps (2010–2100) under two climate change scenarios (SSP1‐2.6 and SSP5‐8.5), assuming a dispersal rate of 200 m/year. Dots indicate mean values calculated across multiple binarization thresholds and global circulation models, while whiskers refer to standard deviation values. (a) Total suitable area over time. (b) Total area of AUs spatial projections within the ENM‐predicted suitable areas (see Figure 4d for AU clustering details). Colors represent different AUs (AU1—yellow; AU2—blue; AU3—red). (c) Genetic offset values projected in geographic space within the ENM‐predicted suitable areas of each AU.

**FIGURE A11:**
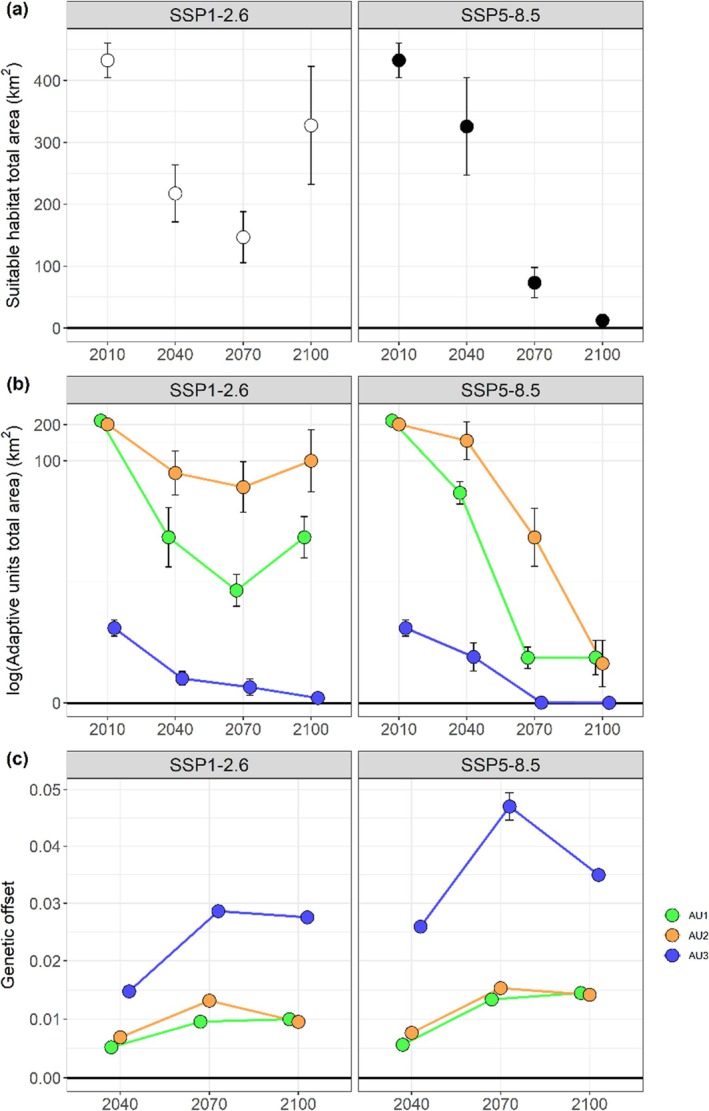
Extent of suitable habitat for *M. bourgati* as predicted by Ecological Niche Models (ENMs), spatial projections of adaptive units (AUs) and genetic offset values, with a 200 m radius buffer around binary suitable patches. Suitable areas were estimated across four‐time steps (2010–2100) under two climate change scenarios (SSP1‐2.6 and SSP5‐8.5), assuming a dispersal rate of 50 m/year. Dots indicate mean values calculated across multiple binarization thresholds and global circulation models, while whiskers refer to standard deviation values. (a) Total suitable area over time. (b) Total area of AUs spatial projections within the ENM‐predicted suitable areas (see Figure 4d for AU clustering details). Colors represent different AUs (AU1—yellow; AU2—blue; AU3—red). (c) Genetic offset values projected in geographic space within the ENM‐predicted suitable areas of each AU.

**FIGURE A12:**
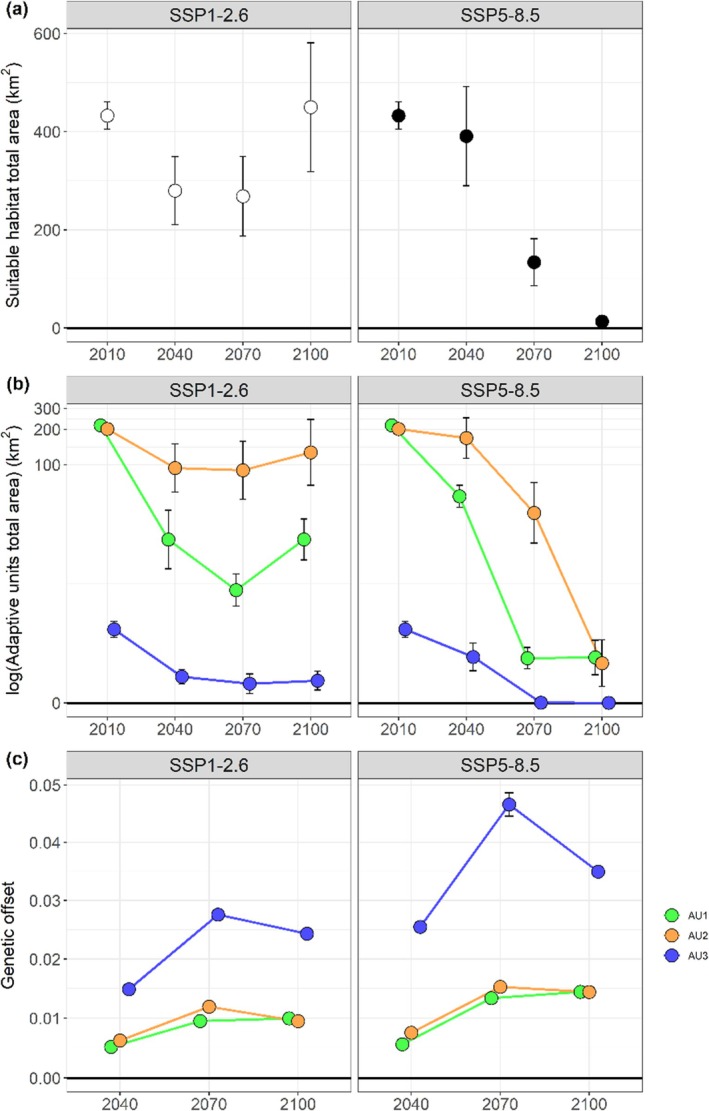
Extent of suitable habitat for *M. bourgati* as predicted by Ecological Niche Models (ENMs), spatial projections of adaptive units (AUs) and genetic offset values, with a 200 m radius buffer around binary suitable patches. Suitable areas were estimated across four‐time steps (2010–2100) under two climate change scenarios (SSP1‐2.6 and SSP5‐8.5), assuming a dispersal rate of 100 m/year. Dots indicate mean values calculated across multiple binarization thresholds and global circulation models, while whiskers refer to standard deviation values. (a) Total suitable area over time. (b) Total area of AUs spatial projections within the ENM‐predicted suitable areas (see Figure 4d for AU clustering details). Colors represent different AUs (AU1—yellow; AU2—blue; AU3—red). (c) Genetic offset values projected in geographic space within the ENM‐predicted suitable areas of each AU.

**FIGURE A13:**
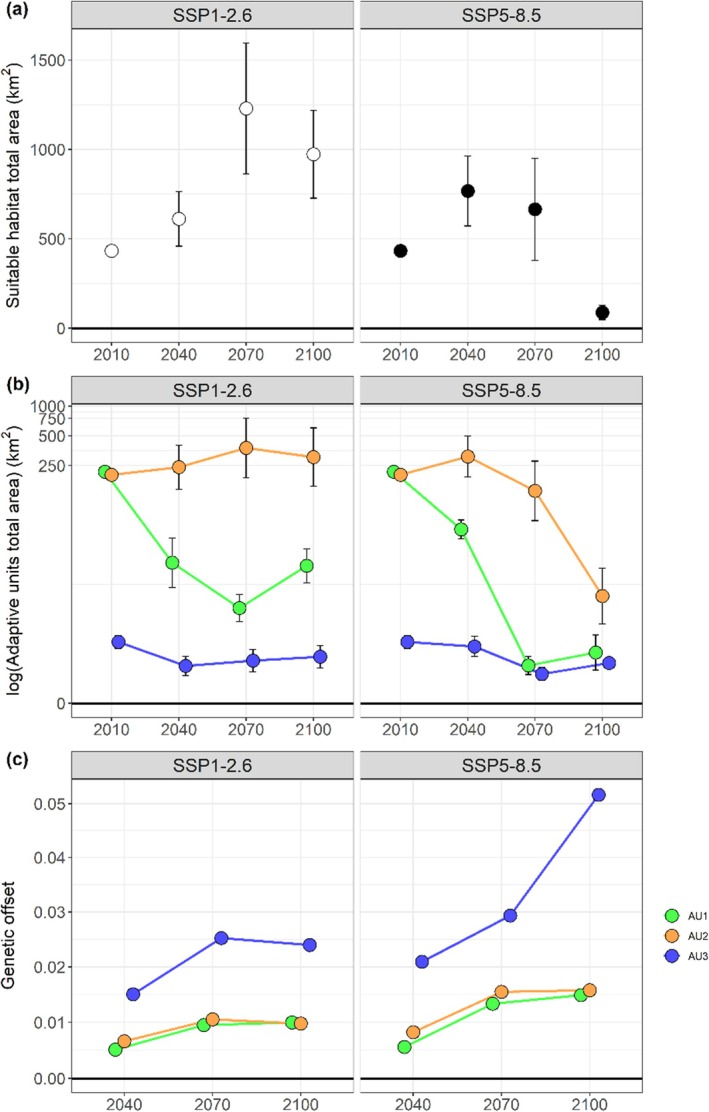
Extent of suitable habitat for *M. bourgati* as predicted by Ecological Niche Models (ENMs), spatial projections of adaptive units (AUs) and genetic offset values, with a 200 m radius buffer around binary suitable patches. Suitable areas were estimated across four‐time steps (2010–2100) under two climate change scenarios (SSP1‐2.6 and SSP5‐8.5), assuming a dispersal rate of 200 m/year. Dots indicate mean values calculated across multiple binarization thresholds and global circulation models, while whiskers refer to standard deviation values. (a) Total suitable area over time. (b) Total area of AUs spatial projections within the ENM‐predicted suitable areas (see Figure 4d for AU clustering details). Colors represent different AUs (AU1—yellow; AU2—blue; AU3—red). (c) Genetic offset values projected in geographic space within the ENM‐predicted suitable areas of each AU.

**FIGURE A14:**
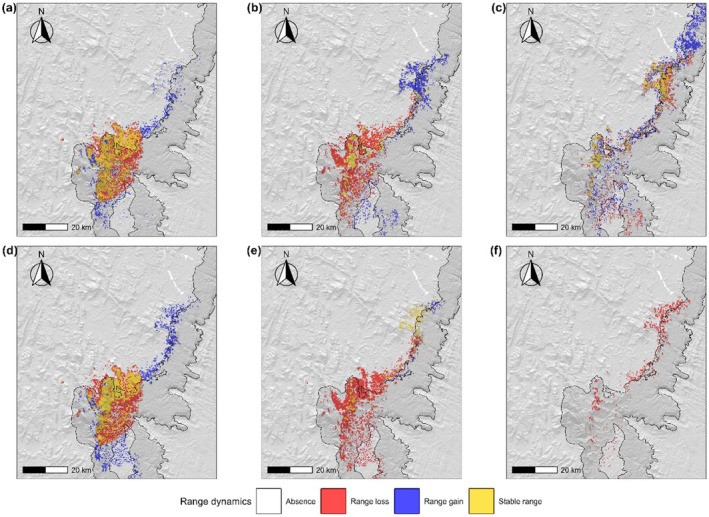
Temporal dynamics in habitat suitability for *M. bourgati* as predicted by Ecological Niche Models (ENMs) from 2010 to 2100 under two climate change scenarios, with a 200 m radius buffer around binary suitable patches. Panels (a–c) show projections under SSP1‐2.6, while panels (d–f) show projections under SSP5‐8.5. Time intervals correspond to: (a, d) 2010–2040; (b, e) 2040–2070; (c, f) 2070–2100. Projections depicted in the figure are based on the 100 m/year dispersal scenario and were generated using the maximize TSS binarization threshold and the IPSL–CM6A–LR global circulation model. Protected area boundaries are outlined in black and filled with a darker gray tone (see Figure 1 for details).

Between 2040 and 2070, models projected a pronounced reduction in the whole extent of suitable areas (Figure 3b,e). Finally, between 2070 and 2100 under SSP1‐2.6, most of the gained suitable area is projected towards the northeast, indicating a potential range shift of suitable habitats approximately 20–30 km beyond the species' current distribution and 45 km from the Andringitra Massif (Figure 3c). The corresponding figure for SSP5‐8.5 suggests an almost complete loss of suitable habitat for the species in the study area (Figure 3f). This general pattern is overall consistent across all three dispersal scenarios, with the only exception emerging under the 200 m/year case, where the species' potential distribution is predicted to remain substantially stable under the SSP1‐2.6 scenario (Figures A9 and A10). For each dispersal scenario, these temporal patterns remained consistent when applying a 200 m radius buffer around binary suitable patches, although the overall extent of suitable areas was larger (Figures A11, A12, A13), as also reflected in the spatial projections, which indicated more pronounced dynamics of potential range gain, loss, and maintenance (Figure A14).

### 3.2 Population Genetic Structure and Isolation‐By‐Distance

**FIGURE A15:**
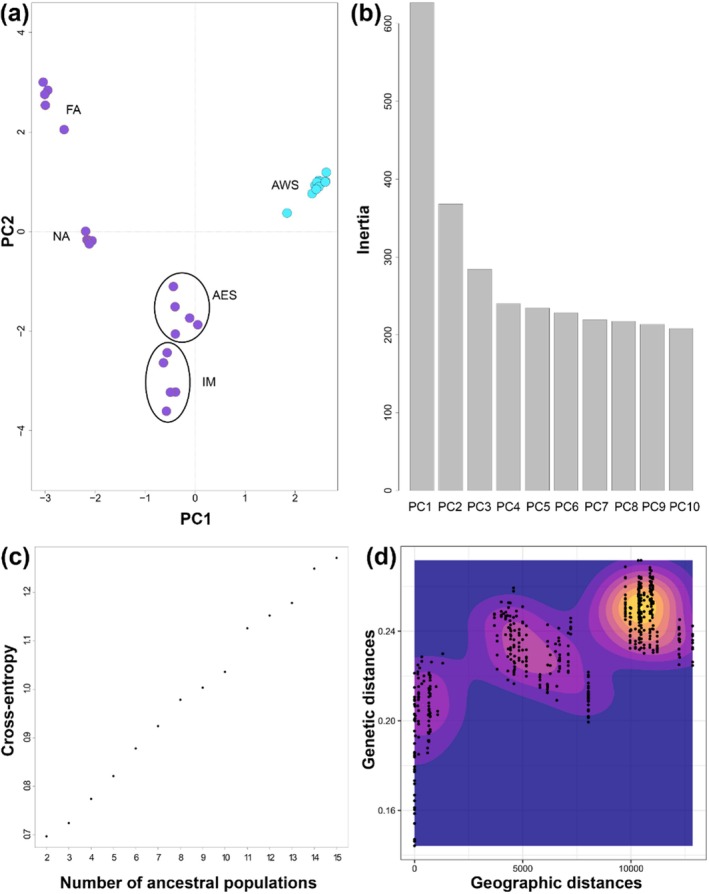
Population genetic structure of *M. bourgati* from SNP genotypes. (a) PC1 vs. PC2 scatterplot of Principal Component Analysis (PCA); individuals are colored based on the two genetic clusters identified with the sparse nonnegative matrix factorization (sNMF) method (see Figure 4a,b; ellipses distinguish individuals from Andringitra Eastern Slopes and Imaitso populations). (b) Screeplot showing the inertia of the first PCA principal components. (c) sNMF cross‐entropy scores averaged over 10 replicates for each value of *K* (number of ancestral populations), used to determine the optimal number of clusters. (d) Correlation between individual genetic and geographic distances: Two‐dimensional kernel density estimation of individual pairwise comparisons between genetic and geographic distances, with black dots representing individual pairwise comparisons and color contours indicating the density of points (darker regions representing lower density and lighter regions representing higher density). See Figure 1 for population distributions. Populations codes: AES, Andingitra Eastern Slopes; AWS, Andringitra Western Slopes; FA, Fivahona Ambavanala; IM, Imaitso; NA, Namoly.

The first two principal components (PCs) explained 9.8% and 5.8% of the total genetic variance, respectively. The PC1 vs. PC2 scatterplot suggested a genetic differentiation related to population geographic distribution along both axes. Along the PC1 axis, the western population (AWS) separated from all others occurring on the eastern side of the study region and the Andringitra Massif (Figure A15a,b), while PC2 differentiated eastern populations.

**FIGURE 4:**
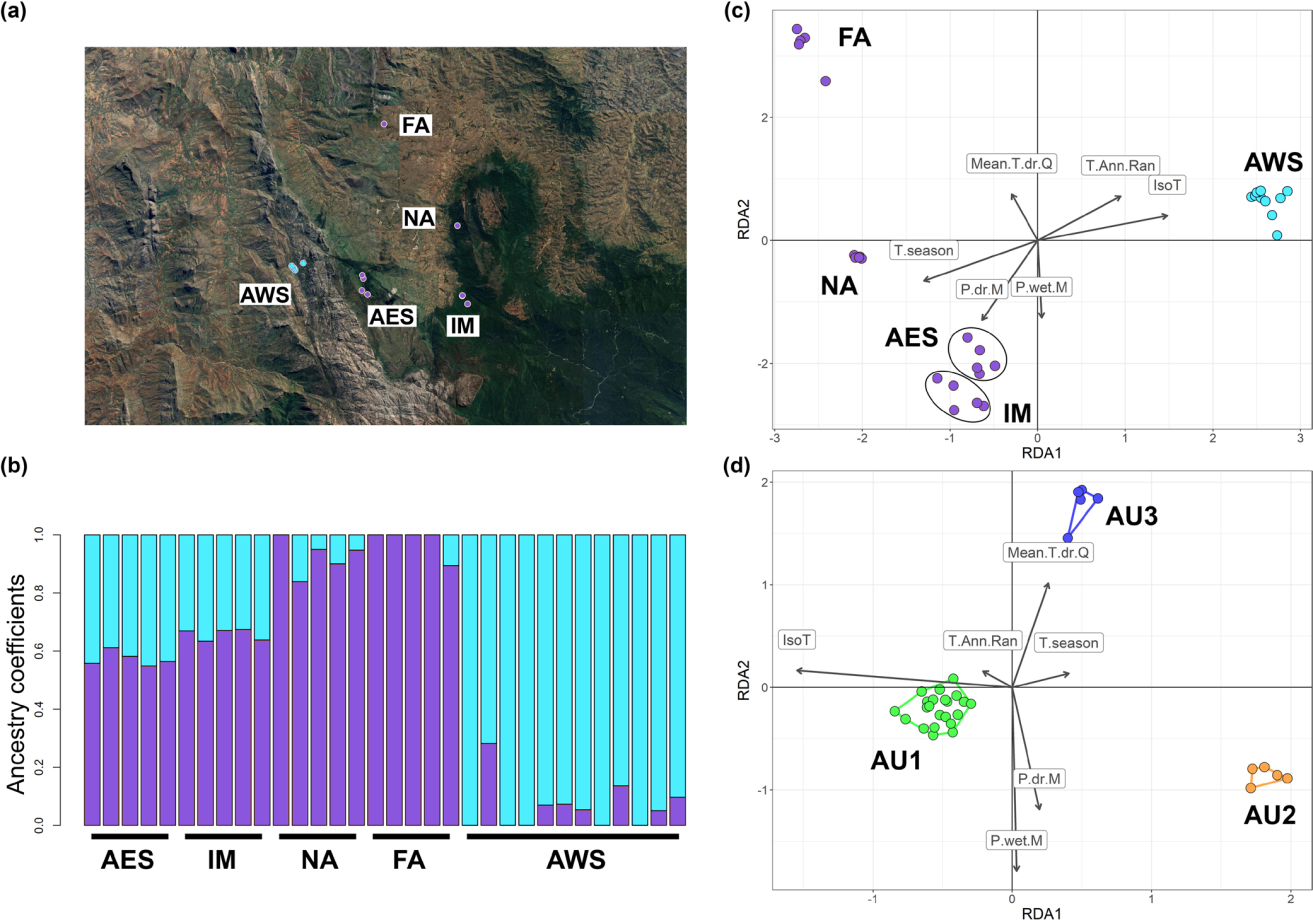
Population genetic structure and adaptive landscape of *M. bourgati* from SNP individual genotype data. (a) Geographic distribution across the study area of the two genetic clusters identified with the sparse nonnegative matrix factorization (sNMF) analysis; individuals are colored according to the dominant genetic cluster to which they were assigned, defined by an ancestry coefficient exceeding 50%. (b) Barplot of individual ancestry coefficients for the selected *K* (= 2) clusters, with each vertical bar representing one individual and colors indicating ancestry proportions from each inferred cluster. (c) RDA performed on the full SNP dataset: RDA1 vs. RDA2 scatterplot with individuals colored based on the two genetic clusters identified with the sNMF method (ellipses highlight and distinguish individuals from the AES and IM populations which show substantial admixture with the western cluster). (d) RDA performed on the 58 SNPs putatively under selection and adaptive units (AUs) clustering through random forest; AU color coding: AU1 (populations AWS, AES, and IM)—yellow; AU2 (NA)—blue; AU3 (FA)—red. Populations are coded as follows: AES, Andringitra Eastern Slopes; AWS, Andringitra Western Slopes; FA, Fivahona Ambavanala; IM, Imaitso; NA, Namoly.

Based on the cross‐entropy criterion, the optimal number of ancestral populations identified by the sNMF analysis was *K* = 2 (Figure A15c). The two genetic clusters displayed a west–east structuring (Figure 4a), closely reflecting the pattern observed in the PCA: the western cluster comprised individuals from the AWS population, while the eastern cluster included those from Andringitra Eastern Slopes (AES), Imaitso (IM), Namoly (NA), and Fivahona Ambavanala (FA) populations. Despite their higher affinity with the eastern cluster, the populations AES and IM displayed substantial admixture with the western cluster, as suggested by their individual ancestry coefficients (Figure 4b). The Mantel test displayed a significant positive correlation between genetic and geographic distances at the individual level (Mantel *r* = 0.7232, *p*‐value = 0.001), suggesting a pattern of IBD in the investigated populations (Figure A15d).

### 3.3 Loci Under Selection

LFMM analyses identified 346 candidate SNPs associated with one or more environmental variables. The number of candidates detected for each variable was as follows: isothermality (123), temperature seasonality (98), temperature annual range (42), mean temperature of driest quarter (53), precipitation of wettest month (97), and precipitation of driest month (60). The three retained RDA axes explained 34.10%, 19.41%, and 13.86% of the total constrained inertia, respectively (Figure A5a). Using these three axes, we identified 172 SNPs associated with bioclimatic variables with the following numbers: isothermality (77), temperature seasonality (15), temperature annual range (3), mean temperature of driest quarter (2), precipitation of wettest month (55), and precipitation of driest month (20). The RDA1 vs. RDA2 scatterplot suggested a clear differentiation between populations on the western and eastern sides of the Andringitra Massif along RDA1, as well as further separation among eastern populations along RDA2 (Figure 4c). Most populations were associated with specific environmental variables. For example, individuals from the AWS population were mostly associated with isothermality and temperature seasonality, those from FA with mean temperature of driest quarter, and populations from AES and IM with precipitation of driest month. The RDA1 vs. RDA3 scatterplot (Figure A5b) provided additional insight, with RDA3 axis further differentiating the NA population from the rest of eastern populations and suggesting some within‐population differentiation in the AWS, along with an association to temperature annual range variable. Overall, we retained 58 unique candidate SNPs jointly identified as outliers by both LFMM and RDA analyses (ca. 0.09% of the starting 6381 SNPs).

### 3.4 Adaptive Units

**FIGURE A16:**
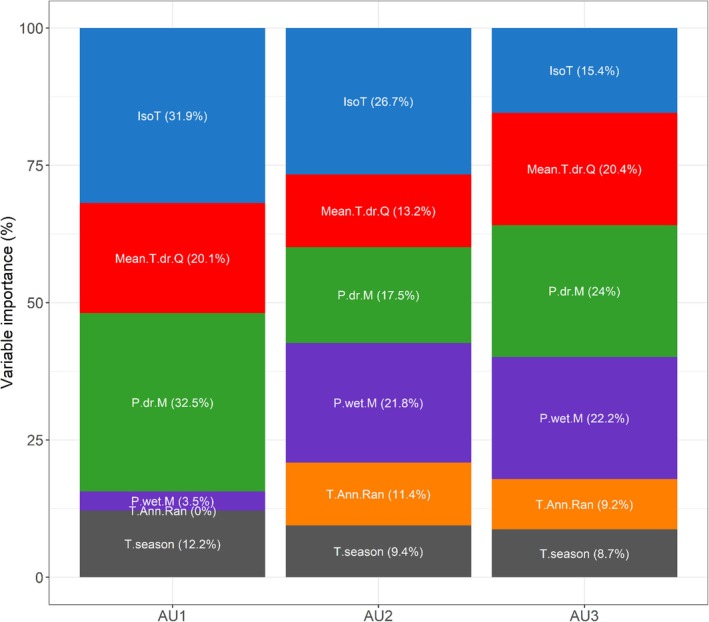
Variable importance scores (measured by the Gini index) for the three adaptive units (AUs) identified through the random forest classification model. For each AU, the relative contribution of the six bioclimatic predictors is expressed as a percentage of the total Gini importance across all variables. See Table S3 for bioclimatic predictor description.

The clustering procedure identified three AUs as the clustering solution most supported by the data (Figure 4d; Text S3; Table S5). These three AUs correspond to the following populations: AWS, AES, and IM (AU1); NA (AU2); FA (AU3). RF classification model reported a high classification performance, achieving a classification accuracy value = 0.88 (SD = 0.19). Variable importance scores indicated precipitation of driest month and isothermality as the most important predictors characterizing AU1, isothermality and precipitation of wettest month characterizing AU2, and precipitation of driest month, precipitation of wettest month and mean temperature of driest quarter discriminating AU3 (Figure A16). The associations between AUs and climatic predictors appear to reflect the environmental conditions characterizing the areas they inhabit. AU1 was positively associated with isothermality, suggesting that populations from the slopes of the Andringitra Massif are associated with climatic conditions characterized by large diurnal temperature variation relative to annual variation, which is generally low (Figure 4d). The negative association of AU2 with isothermality, along with the positive association with precipitation of wettest month, suggests that the NA population is associated with more stable environments, characterized by high precipitations and a low ratio between day/night vs. annual temperature range (Figure 4d). AU3 was positively associated with mean temperature of driest quarter and inversely associated with precipitation in the driest and wettest months, suggesting that the FA population is related to more xeric environments (Figure 4d).

**FIGURE 5:**
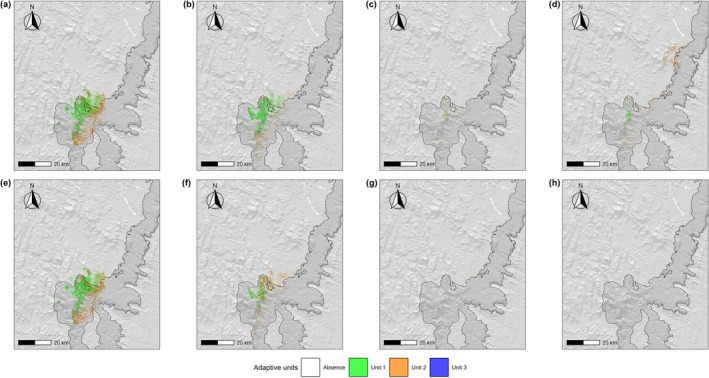
Spatial projections of *M. bourgati* adaptive units (AUs) within habitat suitability areas as predicted by Ecological Niche Models (ENMs) from 2010 to 2100 under two climate change scenarios (see Figure 4d for AU clustering details). Panels (a–d) show projections under SSP1‐2.6, while panels (e–h) show projections under SSP5‐8.5. Time steps correspond to: (a, e) 2010; (b, f) 2040; (c, g) 2070; (d, h) 2100. Projections depicted in the figure are based on the 100 m/year dispersal scenario and were generated using the maximize TSS binarization threshold and the IPSL–CM6A–LR global circulation model. Protected area boundaries are outlined in black and filled with a darker gray tone (see Figure 1 for details).

**FIGURE A17:**
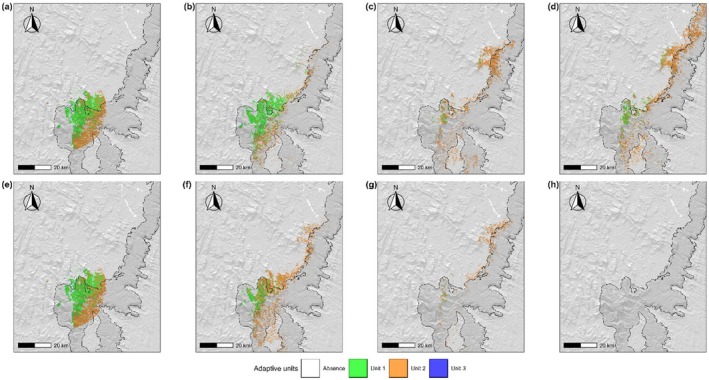
Spatial projections of *M. bourgati* adaptive units (AUs) within habitat suitability areas as predicted by Ecological Niche Models (ENMs) from 2010 to 2100 under two climate change scenarios, with a 200 m radius buffer around binary suitable patches (see Figure 4d for AU clustering details). Panels (a–d) show projections under SSP1‐2.6, while panels (e–h) show projections under SSP5‐8.5. Time steps correspond to: (a, e) 2010; (b, f) 2040; (c, g) 2070; (d, h) 2100. Projections depicted in the figure are based on the 100 m/year dispersal scenario and were generated using the maximize TSS binarization threshold and the IPSL–CM6A–LR global circulation model. Protected area boundaries are outlined in black and filled with a darker gray tone (see Figure 1 for details).

ENM projections predict a general decline in the area potentially occupied by all AUs from 2010 to 2100, with relevant differences between AUs, climate change scenarios (Figure 2b), and, in some cases, dispersal scenarios (Figures A9 and A10). The suitable area for AU3 is already extremely limited under current conditions and is projected to further diminish and disappear by 2070 under SSP1‐2.6, or by 2040 under SSP5‐8.5. AU1 currently occupies a slightly larger suitable area (approximately 100 km^2^) than AU2, but AU2 is expected to experience a slower reduction in suitable area compared to AU1, ultimately projected to become the dominant AU across the species' range. Under SSP1‐2.6, both units are projected to experience a slight increase in suitable area between 2070 and 2100, reaching approximately 50 km^2^. In contrast, under SSP5‐8.5, suitable areas for both units are projected to continue declining steadily, approaching zero by the end of the century (Figure 2b). At present and by 2040, AU1 is projected to be the most widespread unit across areas overlapping the species' current distributional range, while AU2 is primarily projected in more peripheral regions (Figure 5a,b,e,f). Between 2070 and 2100, under SSP1‐2.6—where the level and distribution of suitable areas remain more appreciable—the geographical separation between the two AUs becomes more pronounced, with the suitable area of AU1 persisting near the current distribution and the suitable area of AU2 becoming dominant in areas to the northeast, where most of the species' suitable habitat is projected by 2100 (Figure 5c,d). A comparable pattern emerged when projections were generated using a 200 m radius buffer around binary suitable patches, although with a larger extent of suitable areas (Figures A11, A12, A13 and A17). Under this buffering approach, AU3 was projected to retain some suitable habitat for a longer period, although predicted values remained close to zero (Figures A11, A12, A13 and A17), and spatial projections indicated more pronounced dynamics of potential range gain, loss, and maintenance for each AU (Figure A17).

### 3.5 Genetic Offset

**FIGURE 6:**
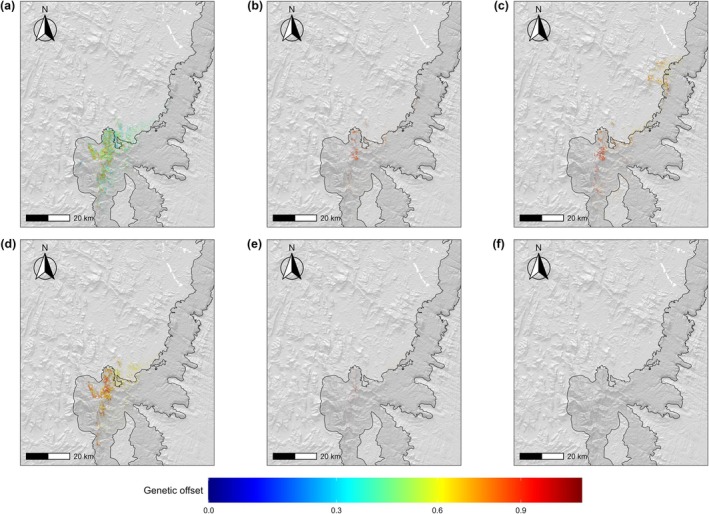
Spatial projections of *M. bourgati* genetic offset values within habitat suitability areas as predicted by Ecological Niche Models (ENMs) from 2010 to 2100 under two climate change scenarios. Panels (a–c) show projections under SSP1‐2.6, while panels (d–f) show projections under SSP5‐8.5. Time intervals correspond to: (a, d) 2010–2040; (b, e) 2040–2070; (c, f) 2070–2100. Projections depicted in the figure are based on the 100 m/year dispersal scenario and were generated using the maximize TSS binarization threshold and the IPSL–CM6A–LR global circulation model. Protected area boundaries are outlined in black and filled with a darker gray tone (see Figure 1 for details).

**FIGURE A18:**
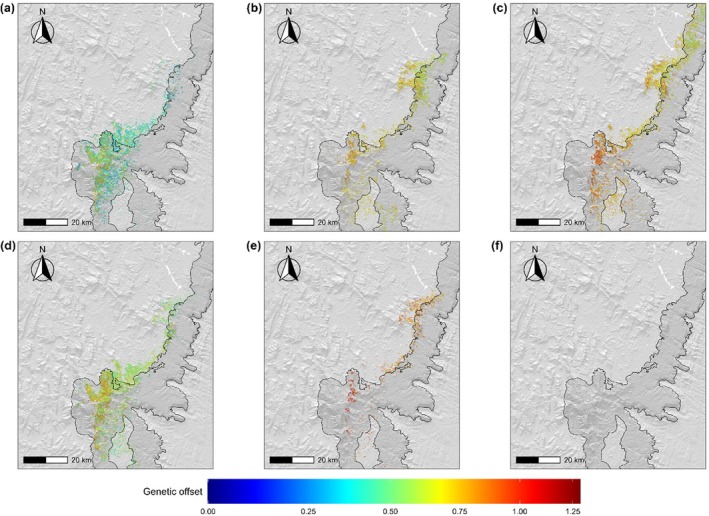
Spatial projections of *M. bourgati* genetic offset values within habitat suitability areas as predicted by Ecological Niche Models (ENMs) from 2010 to 2100 under two climate change scenarios, with a 200 m radius buffer around binary suitable patches. Panels (a–c) show projections under SSP1‐2.6, while panels (d–f) show projections under SSP5‐8.5. Time intervals correspond to: (a, d) 2010–2040; (b, e) 2040–2070; (c, f) 2070–2100. Projections depicted in the figure are based on the 100 m/year dispersal scenario and were generated using the maximize TSS binarization threshold and the IPSL–CM6A–LR global circulation model. Protected area boundaries are outlined in black and filled with a darker gray tone (see Figure 1 for details).

Projections suggested that genetic offset will generally increase for all AUs over the course of the century, with the SSP5‐8.5 scenario predicting consistently higher values than SSP1‐2.6 (Figure 2c). AU1 and AU2 exhibited comparable magnitudes of genetic offset and similar temporal patterns, characterized by a pronounced increase between 2040 and 2070 followed by stabilization until 2100, whereas genetic offset values for AU3 were projected to be considerably higher than those of the other AUs (Figure 2c). These patterns were consistent across all dispersal scenarios (Figures A9 and A10). Between 2010 and 2040, genetic offset values are generally projected to be moderate under SSP1‐2.6 and high under SSP5‐8.5 across most of the suitable areas (Figure 6a,d). From 2040 to 2070, genetic offset is projected to increase to high values within the few remaining suitable areas in both scenarios (Figure 6b,e). Between 2070 and 2100, under SSP1‐2.6 genetic offset is projected to remain high near the current species' range, while moderate values are observed in the northeastern area where suitable habitat is expected to shift (Figure 6c). Results based on the 200 m radius buffer approach predicted broadly comparable temporal patterns, while increasing the spatial extent over which genetic offset was mapped (Figures A11, A12, A13). Under this buffering approach, AU3 represented an exception: owing to a larger predicted habitat suitability, nonzero genetic offset values persisted towards the end of the century. Also, AU3 was predicted to exhibit a temporal pattern similar to that of AU1 and AU2, with genetic offset generally increasing until 2070 followed by stabilization or a slight decrease across dispersal scenarios, except for a sharp increase under the 200 m/year scenario in SSP5‐8.5 (Figures A11, A12, A13 and A18). Genetic offset estimates derived using the GF approach predicted a similar temporal trend (see Text S2 for details; Figures A6 and A7).

### 3.6 Protected Area Coverage of Suitable Habitat, Adaptive Units, and Genetic Offset

**FIGURE 7:**
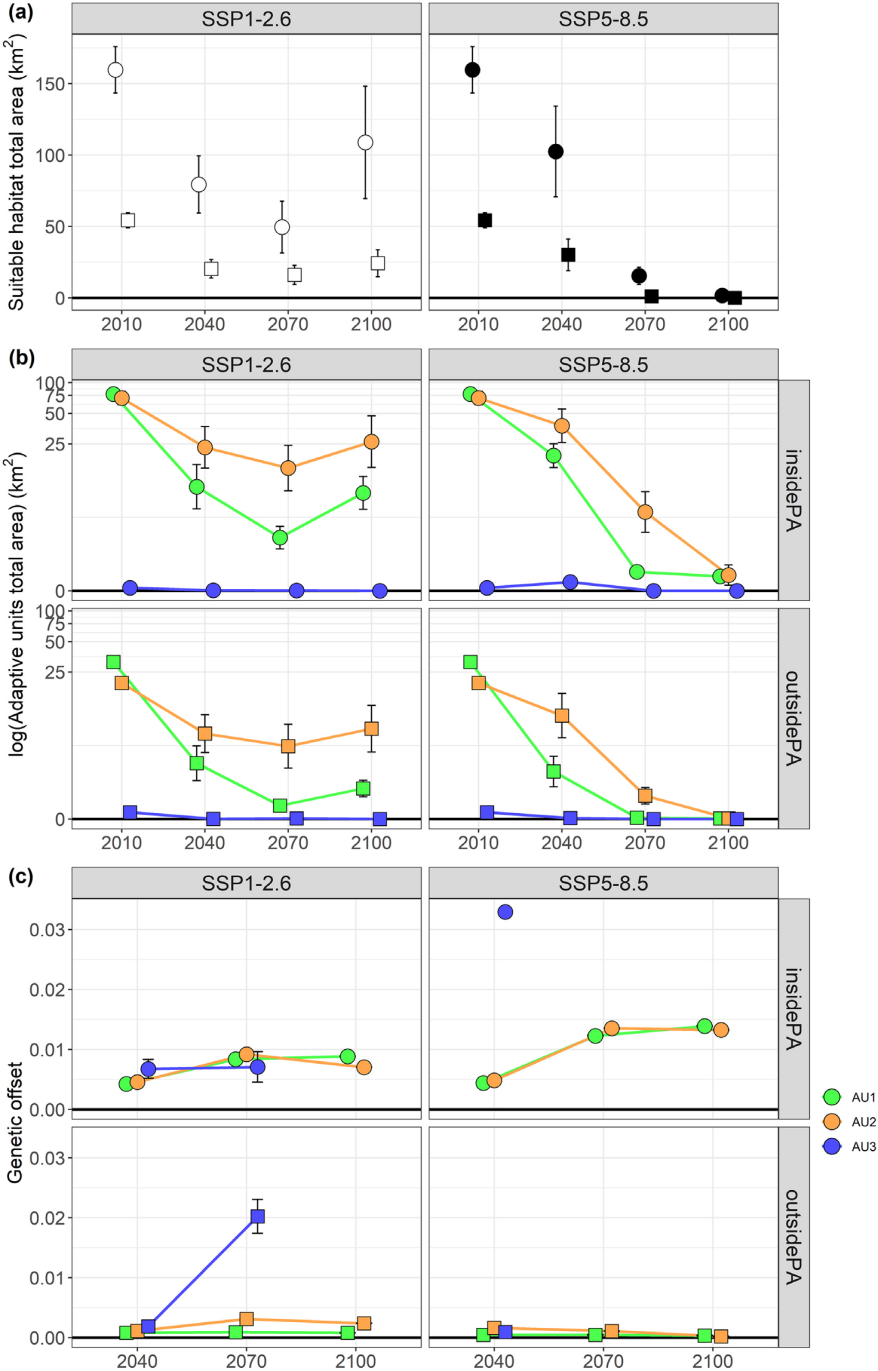
Extent of suitable habitat for *M. bourgati* as predicted by Ecological Niche Models (ENMs), spatial projections of adaptive units (AUs) and genetic offset values, within (circles) and outside (squares) Madagascar's protected area network (PAs). Suitable areas were estimated across four‐time steps (2010–2100) under two climate change scenarios (SSP1‐2.6 and SSP5‐8.5), assuming a dispersal rate of 100 m/year. Circles and squares indicate mean values calculated across multiple binarization thresholds and global circulation models, while whiskers refer to standard deviation values. (a) Total suitable area over time within (circles) and outside (squares) Madagascar's PAs. (b) Total area of AUs spatial projections within the ENM‐predicted suitable areas within (circles) and outside (squares) Madagascar's PAs (see Figure 4d for AU clustering details). Colors represent different AUs (AU1—yellow; AU2—blue; AU3—red). (c) Genetic offset values projected in geographic space within the ENM‐predicted suitable areas within (circles) and outside (squares) Madagascar's PAs. See Figure 1 for PA distribution across the study area.

**FIGURE A19:**
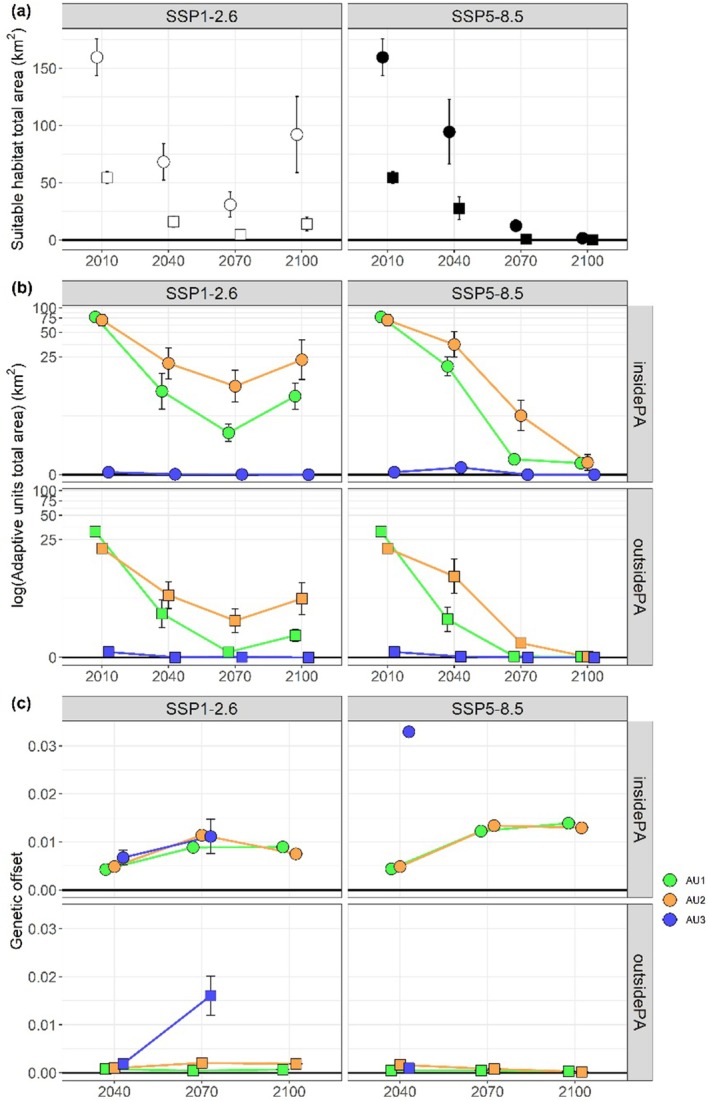
Extent of suitable habitat for *M. bourgati* as predicted by Ecological Niche Models (ENMs), spatial projections of adaptive units (AUs) and genetic offset values, within (circles) and outside (squares) the network of protected areas (PAs) of Madagascar. Suitable areas were estimated across four‐time steps (2010–2100) under two climate change scenarios (SSP1‐2.6 and SSP5‐8.5), assuming a dispersal rate of 50 m/year. Circles/squares indicate mean values calculated across multiple binarization thresholds and global circulation models, while whiskers refer to standard deviation values. (a) Total suitable area over time within (circles) and outside (squares) Madagascar's PAs. (b) Total area of AUs spatial projections within the ENM‐predicted suitable areas within (circles) and outside (squares) Madagascar's PAs (see Figure 4d for AU clustering details). Colors represent different AUs (AU1—yellow; AU2—blue; AU3—red). (c) Genetic offset values projected in the geographic space within the ENM‐predicted suitable areas within (circles) and outside (squares) Madagascar's PAs. See Figure 1 for PAs distribution across the study area.

**FIGURE A20:**
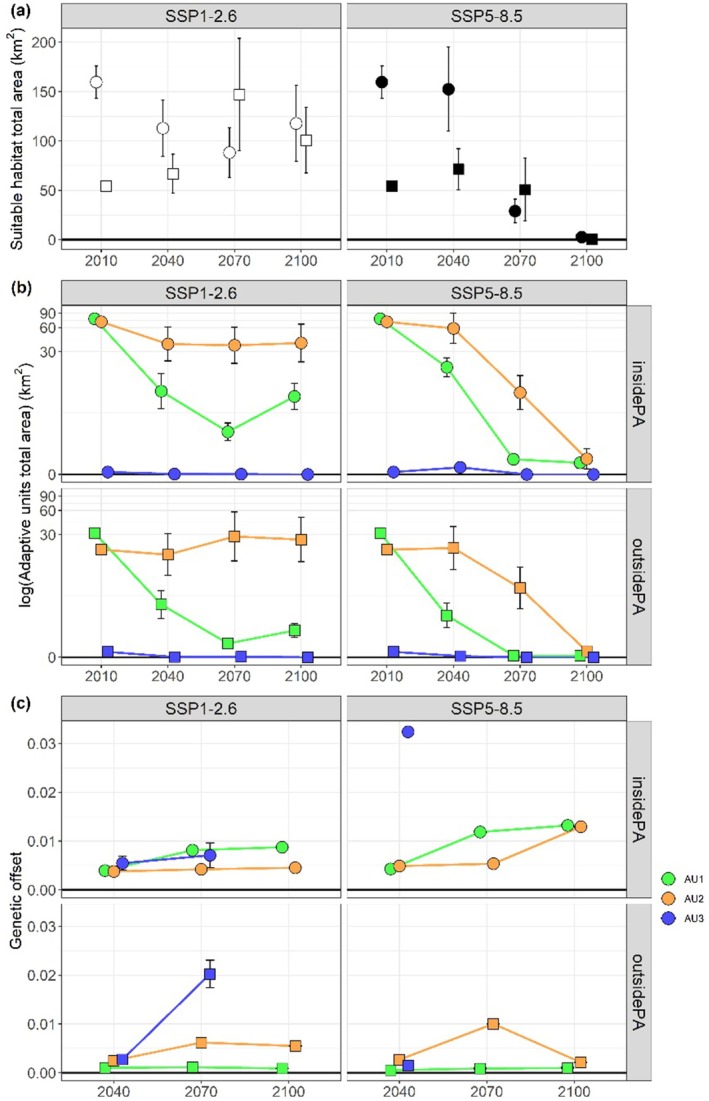
Extent of suitable habitat for *M. bourgati* as predicted by Ecological Niche Models (ENMs), spatial projections of adaptive units (AUs) and genetic offset values, within (circles) and outside (squares) the network of protected areas (PAs) of Madagascar. Suitable areas were estimated across four‐time steps (2010–2100) under two climate change scenarios (SSP1‐2.6 and SSP5‐8.5), assuming a dispersal rate of 200 m/year. Circles/squares indicate mean values calculated across multiple binarization thresholds and global circulation models, while whiskers refer to standard deviation values. (a) Total suitable area over time within (circles) and outside (squares) Madagascar's PAs. (b) Total area of AUs spatial projections within the ENM‐predicted suitable areas within (circles) and outside (squares) Madagascar's PAs (see Figure 4d for AU clustering details). Colors represent different AUs (AU1—yellow; AU2—blue; AU3—red). (c) Genetic offset values projected in the geographic space within the ENM‐predicted suitable areas within (circles) and outside (squares) Madagascar's PAs. See Figure 1 for PAs distribution across the study area.

**FIGURE A21:**
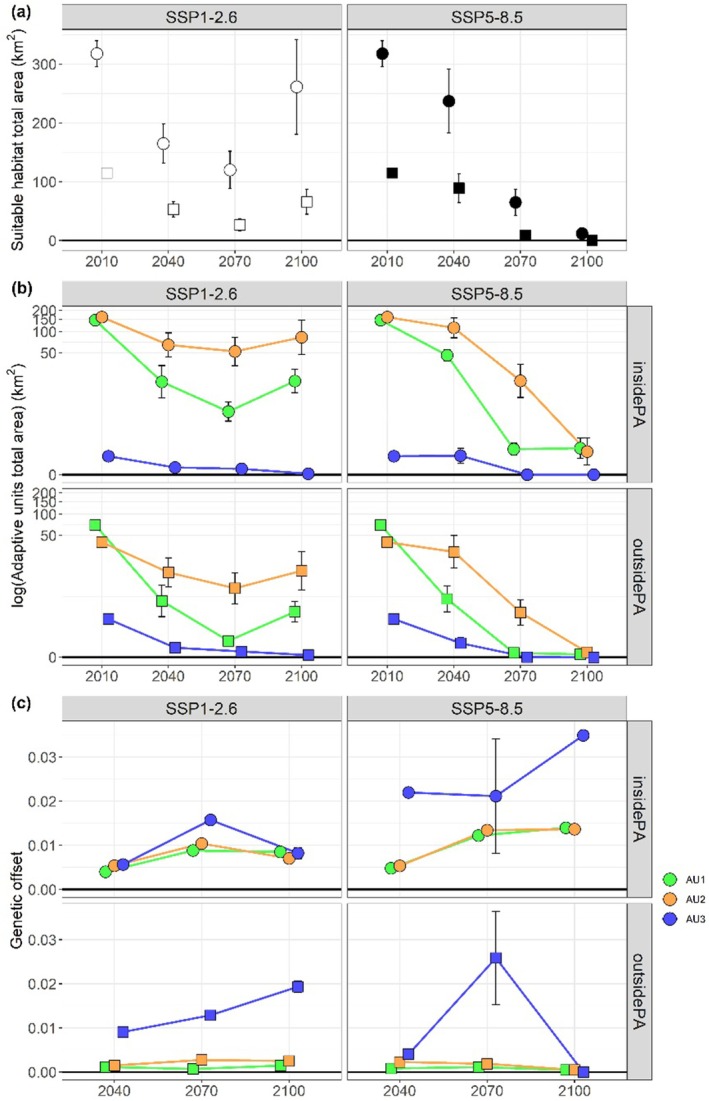
Extent of suitable habitat for *M. bourgati* as predicted by Ecological Niche Models (ENMs), spatial projections of adaptive units (AUs) and genetic offset values, within (circles) and outside (circles) the network of protected areas (PAs) of Madagascar, with a 200 m radius buffer around binary suitable patches. Suitable areas were estimated across four‐time steps (2010–2100) under two climate change scenarios (SSP1‐2.6 and SSP5‐8.5), assuming a dispersal rate of 50 m/year. Circles/squares indicate mean values calculated across multiple binarization thresholds and global circulation models, while whiskers refer to standard deviation values. (a) Total suitable area over time within (circles) and outside (squares) Madagascar's PAs. (b) Total area of AUs spatial projections within the ENM‐predicted suitable areas within (circles) and outside (squares) Madagascar's PAs (see Figure 4d for AU clustering details). Colors represent different AUs (AU1—yellow; AU2—blue; AU3—red). (c) Genetic offset values projected in the geographic space within the ENM‐predicted suitable areas within (circles) and outside (squares) Madagascar's PAs. See Figure 1 for PAs distribution across the study area.

**FIGURE A22:**
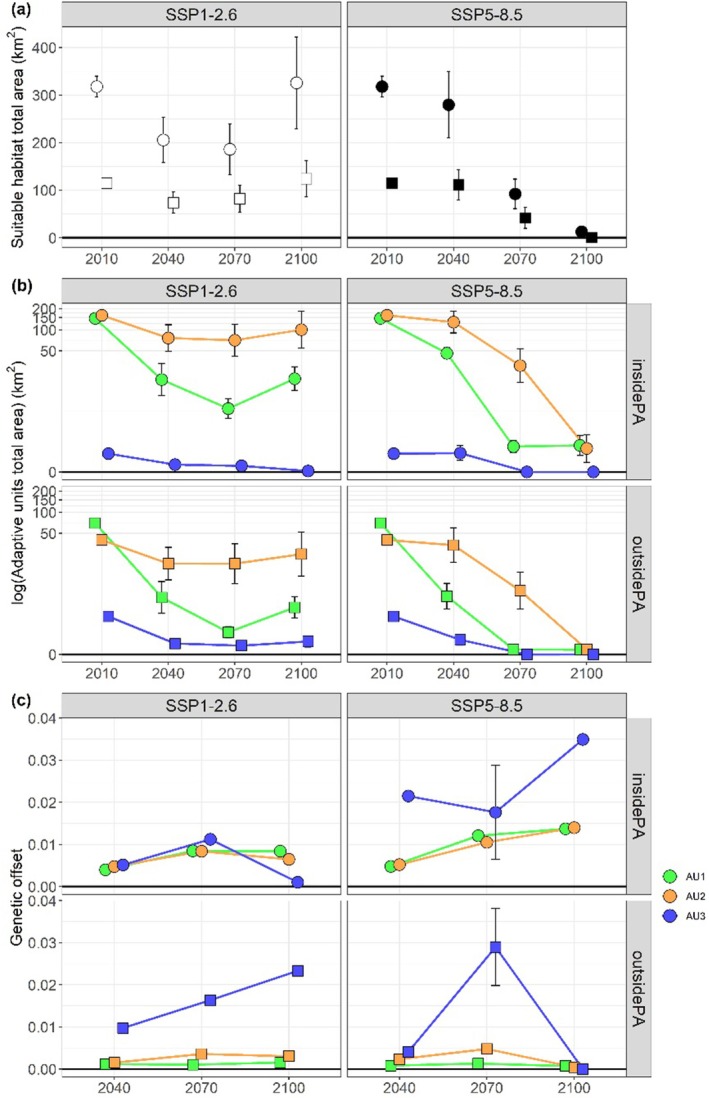
Extent of suitable habitat for *M. bourgati* as predicted by Ecological Niche Models (ENMs), spatial projections of adaptive units (AUs) and genetic offset values, within (circles) and outside (squares) the network of protected areas (PAs) of Madagascar, with a 200 m radius buffer around binary suitable patches. Suitable areas were estimated across four‐time steps (2010–2100) under two climate change scenarios (SSP1‐2.6 and SSP5‐8.5), assuming a dispersal rate of 100 m/year. Circles/squares indicate mean values calculated across multiple binarization thresholds and global circulation models, while whiskers refer to standard deviation values. (a) Total suitable area over time within (circles) and outside (squares) Madagascar's PAs. (b) Total area of AUs spatial projections within the ENM‐predicted suitable areas within (circles) and outside (squares) Madagascar's PAs (see Figure 4d for AU clustering details). Colors represent different AUs (AU1—yellow; AU2—blue; AU3—red). (c) Genetic offset values projected in the geographic space within the ENM‐predicted suitable areas within (circles) and outside (squares) Madagascar's PAs. See Figure 1 for PAs distribution across the study area.

**FIGURE A23:**
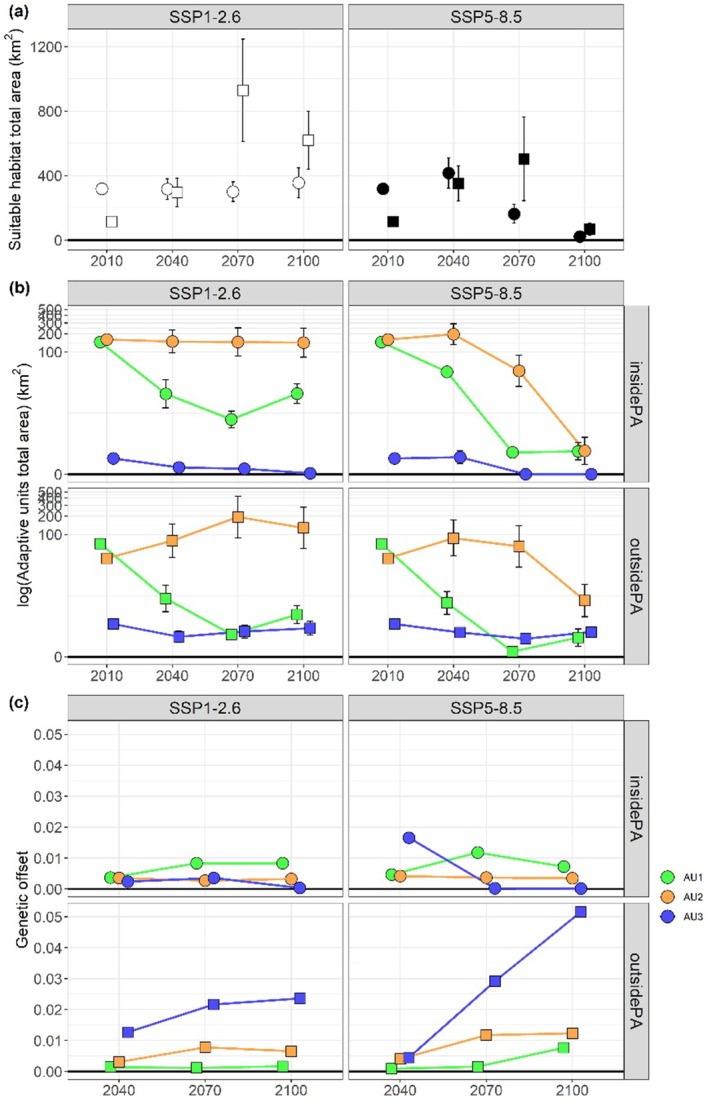
Extent of suitable habitat for *M. bourgati* as predicted by Ecological Niche Models (ENMs), spatial projections of adaptive units (AUs) and genetic offset values, within (circles) and outside (squares) the network of protected areas (PAs) of Madagascar, with a 200 m radius buffer around binary suitable patches. Suitable areas were estimated across four‐time steps (2010–2100) under two climate change scenarios (SSP1‐2.6 and SSP5‐8.5), assuming a dispersal rate of 200 m/year. Circles/squares indicate mean values calculated across multiple binarization thresholds and global circulation models, while whiskers refer to standard deviation values. (a) Total suitable area over time within (circles) and outside (squares) Madagascar's PAs. (b) Total area of AUs spatial projections within the ENM‐predicted suitable areas within (circles) and outside (squares) Madagascar's PAs (see Figure 4d for AU clustering details). Colors represent different AUs (AU1—yellow; AU2—blue; AU3—red). (c) Genetic offset values projected in the geographic space within the ENM‐predicted suitable areas within (circles) and outside (squares) Madagascar's PAs. See Figure 1 for PAs distribution across the study area.

**FIGURE A24:**
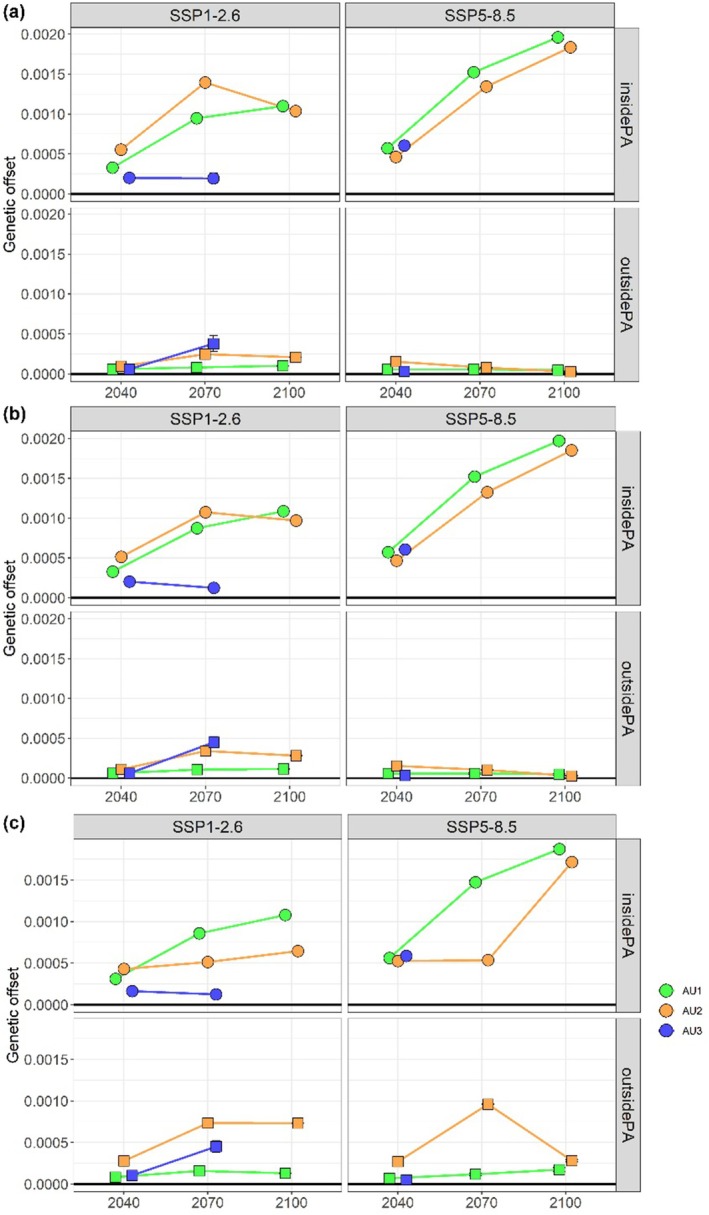
Genetic offset values for *M. bourgati* estimated using the Gradient Forest (GF) approach under two climate change scenarios, within (circles) and outside (squares) the network of protected areas (PAs) of Madagascar. Genetic offset values were projected in geographic space within the Ecological Niche Model‐predicted suitable areas of each adaptive unit (AU) across four‐time steps (2010–2100) under SSP1‐2.6 and SSP5‐8.5 scenarios. Circles/squares indicate mean values calculated across multiple binarization thresholds and global circulation models, while whiskers refer to standard deviation values. (a) Genetic offset assuming a dispersal rate of 50 m/year within (circles) and outside (squares) Madagascar's PAs. (b) Genetic offset assuming a dispersal rate of 100 m/year within (circles) and outside (squares) Madagascar's PAs. (c) Genetic offset assuming a dispersal rate of 200 m/year within (circles) and outside (squares) Madagascar's PAs. See Figure 1 for PAs distribution across the study area.

**FIGURE A25:**
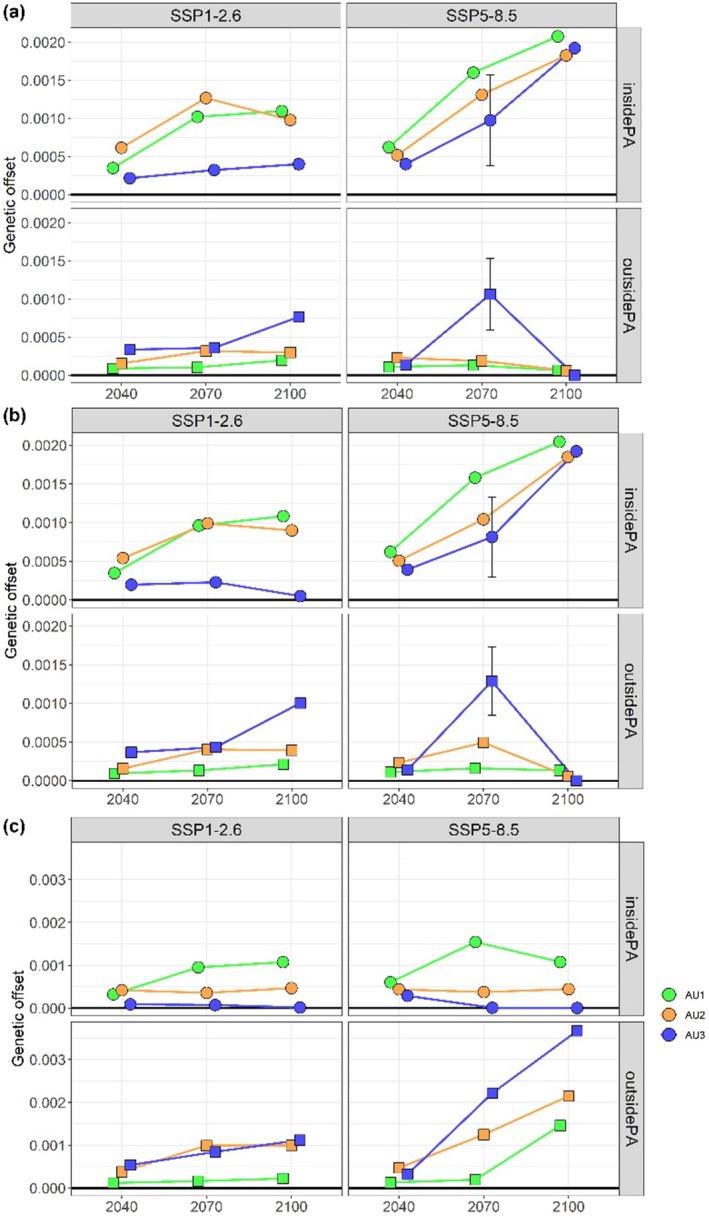
Genetic offset values for *M. bourgati* estimated using the Gradient Forest (GF) approach under two climate change scenarios, within (circles) and outside (squares) the network of protected areas (PAs) of Madagascar. Genetic offset values were projected in geographic space within the Ecological Niche Model‐predicted suitable areas of each adaptive unit (AU) across four‐time steps (2010–2100) under SSP1‐2.6 and SSP5‐8.5 scenarios, with a 200 m radius buffer around binary suitable patches. Circles/squares indicate mean values calculated across multiple binarization thresholds and global circulation models, while whiskers refer to standard deviation values. (a) Genetic offset assuming a dispersal rate of 50 m/year within (circles) and outside (squares) Madagascar's PAs. (b) Genetic offset assuming a dispersal rate of 100 m/year within (circles) and outside (squares) Madagascar's PAs. (c) Genetic offset assuming a dispersal rate of 200 m/year within (circles) and outside (squares) Madagascar's PAs. See Figure 1 for PAs distribution across the study area.

Across both climate change scenarios, the proportion of suitable habitat, both general (Figure 7a) and for each AU (Figure 7b), is projected to consistently remain higher within Madagascar's PA network compared to unprotected regions. However, the extent of suitable habitat outside PAs is projected to increase proportionally over time, along with the general decline in habitat suitability projected from 2010 to 2100 (Figure 7a,b). Notably, genetic offset values are projected to be consistently higher within the PA network than outside it, across all future time steps and climate change scenarios (Figure 7c). This pattern was also evident under the 50 m/year dispersal scenario, whereas under the 200 m/year scenario genetic offset values within and outside PAs were more similar (Figures A19 and A20). Comparable results were obtained using the 200 m radius buffering approach (Figures A21, A22, A23) and when genetic offset was estimated using the GF method under both binarization strategies (Figures A24 and A25).

## 4 Discussion

By integrating ENMs with GEA analyses, we could account for two fundamental mechanisms underlying *M. bourgati* response to future climate change—dispersal and genetic adaptation—thus providing a more comprehensive understanding of the predicted species' vulnerability. ENMs projected a progressive decline in suitable habitat from the present to the end of the century, resulting either in a complete disappearance or a pronounced reduction of suitable habitats, depending on the different climate change scenarios. GEA analyses suggested patterns of genotype‐environment associations consistent with local adaptation, with the identification of three AUs associated with distinct local climatic conditions, which are predicted to respond differently under future climate change. In addition, by explicitly accounting for species' dispersal potential, genetic adaptive capacity and genetic offset, we forecast an alarming future scenario in which, even under the most optimistic climate change projections, the few remaining suitable areas are predicted to be associated with high maladaptation risk. Finally, such a risk is predicted to be higher within the current PA network, further exacerbating species' vulnerability under climate change.

### 4.1 Predicted Changes in Climatically Suitable Areas

Under the most pessimistic climate change scenario (SSP5‐8.5), models project that the progressive decline in the species' suitable habitat until its total extinction will be limited to the high‐elevation region of the Andringitra Massif, where *M. bourgati* is currently present, with no emergence of suitable habitat in the adjacent low‐elevation areas (Figure 3). These projections support the general notion that montane species are particularly vulnerable to climate change (Elsen and Tingley 2015; Mata‐Guel et al. 2023; Raxworthy et al. 2008). These species are expected to track their climatic optima through upslope range shifts, a process that often results in substantial reductions in range size and may result in local extirpation or complete extinction (Elsen and Tingley 2015; Mata‐Guel et al. 2023; Raxworthy et al. 2008). This pattern is especially pronounced in tropical montane forest biotas, where the rate of upslope shifts exceeds that observed in temperate regions (Freeman et al. 2021; Mata‐Guel et al. 2023). In Madagascar, this pattern has been documented on the Tsaratanana Massif, where upslope shifts of up to 51 m have been recorded for the local amphibian and reptile community between 1993 and 2003 (Raxworthy et al. 2008). Similarly, ENM studies predicted that suitable habitats for Malagasy baobab species and for some frogs of the genus *Mantella* will shift to higher elevations under future climate scenarios (by up to 351 m in the case of baobabs) (Carné, Vieites, and Sillero 2025; Tagliari et al. 2021).

Under the optimistic SSP1‐2.6 scenario, suitable habitat is projected to slightly increase between 2070 and 2100, exceeding 2040 extents but remaining markedly below present‐day levels (Figure 2a), a pattern also reported in other amphibians (Boyer et al. 2025). This likely reflects dispersing propagules that, after 2070 in our dispersal simulations, gradually reach suitable patches that were distant and inaccessible during earlier time intervals. Under this scenario, models projected that by 2100 patches of climatically suitable habitat would persist near the high‐elevation areas of the Andringitra Massif, although at lower elevations than the species' current locations (Figure 3). A few suitable areas are also expected to shift northeastward into the low‐elevation forest corridor of Ambositra–Vondrozo (approximately 1000–1200 m a.s.l.), while most suitable areas are projected to occur along a small mountain chain at the northeastern margin of this forest corridor. These mountains, with elevations of up to approximately 1800 m a.s.l., are located approximately 45 km north of the Andringitra Massif, close to the town of Ambohimahamasina (Figure 3c). The Andringitra Massif region and Ambohimahamasina mountains are projected to be separated by a stretch of mostly unsuitable habitat, resulting in a potential highly fragmented and spatially disjunct distribution of suitable areas (Figure 3c). This projected range shift towards the north (i.e., lower latitudes) contrasts with the common trend of poleward movements typically observed in response to climate change (Lenoir and Svenning 2013) and aligns with ENM predictions for some species of Malagasy *Mantella* frogs (Carné, Vieites, and Sillero 2025). A similar northward and downslope shift into forest corridors has been specifically predicted for the microendemic *Mantella aurantiaca* frogs from central Madagascar, although this frog currently reaches up to 1000 m a.s.l., therefore spanning a lower elevational gradient than *M. bourgati* (Edwards et al. 2022).

### 4.2 Genetic Adaptive Capacity

The detected genotype‐environment associations related to climatic variation in populations of *M. bourgati* are interpreted here as proxies of local adaptation, reflecting statistical relationships between allele frequencies and climatic gradients rather than direct evidence of fitness. Within this framework, the observed patterns are consistent with the presence of putative local adaptation. We acknowledge, however, that the GEA approaches adopted here (LFMM and RDA) primarily capture linear genotype‐environment associations and may therefore be less sensitive to complex nonlinear relationships. As a result, the patterns of local adaptation inferred in this study should be interpreted as reflecting the dominant linear components of genotype‐environment covariation. In addition, our analyses focus on SNP variation and do not account for other forms of genetic variation, such as structural variants or copy‐number variation, which may also contribute to local adaptation in natural populations but remain inaccessible using reduced‐representation sequencing approaches such as ddRAD.

The adaptive landscape of *M. bourgati* appears to reflect the environmental characteristics of the habitats occupied by the three identified AUs, suggesting that putative local adaptation is mostly related to temporal variability of temperature and precipitations (Figure 4d). Specifically, the IM, AES, and AWS populations clustered within AU1, suggesting shared association with similar climatic conditions despite their geographic and topographic separation (Figure 4a,d). Their association with high isothermality aligns with the tropical montane environments of the Andringitra Massif where these populations occur (Ghalambor 2006). Similarly, the association of NA population (AU2) with low isothermality and high precipitations during the rainiest season is consistent with its location in mid‐elevation rainforest. Finally, in the FA population, which formed AU3, a distinct association with generally higher temperatures and lower precipitations was predicted, reflecting the warmer and more xeric conditions of this part of the region, where forest cover has been almost entirely lost. Although the species was sampled in all localities where it is confidently known to occur (Table S1), it cannot be excluded that it may also be present in other surrounding areas (Figure 1). Consequently, additional genotype‐environment associations contributing to the species' adaptive landscape may exist but are not captured in the present analyses.

Species' responses to environmental and climatic changes are often heterogeneous among conspecific populations, due to intraspecific variation in genetic and phenotypic traits (Arietta and Skelly 2021; Capblancq et al. 2020; Forester et al. 2022, 2025). While local adaptation was considered a process occurring predominantly at broad spatial scales, a growing body of literature has demonstrated that it can also arise at microgeographic scales (Arietta and Skelly 2021; Forester et al. 2025; Mikles et al. 2020; Richardson et al. 2014). At such scales, local adaptation is assumed to occur when the strength of selection exceeds the homogenizing effects of the high gene flow typically expected at such spatial scales (Richardson et al. 2014; Tigano and Friesen 2016). In our study system, the sNMF analysis of population genetic structure, along with the detection of a significant pattern of IBD (Figures 4a,b and A15d), suggest a continuous pattern of genetic variation structured along a west–east geographic gradient, consistent with the presence of gene flow across the study area. The Andringitra Massif likely represents a permeable barrier allowing individual movements across high‐elevation corridors, as suggested by admixture signatures between the western cluster (AWS population) and the two populations of the eastern cluster closest to the Massif (AES and IM). This is consistent with the species' known elevation range, up to 2500 m a.s.l., close to the Massif highest elevation of 2658 m a.s.l. (Goodman 1996; Guibé 1974; Nicoll and Langrand 1989).

The apparent strength of putative local adaptation inferred in *M. bourgati* populations, which apparently exceeds gene flow, may result from the highly heterogeneous environmental conditions of the Andringitra Massif region (Goodman 1996; Nicoll and Langrand 1989). This area not only presents a pronounced elevation gradient but is also located at the boundary between the eastern rainforest belt and the subhumid forest bioclimatic zones (Goodman 1996; Nicoll and Langrand 1989). The heterogeneous and often extreme environmental conditions characterizing montane ecosystems can impose strong selective pressures that promote local adaptation to these environments (Forester et al. 2025; Giska et al. 2022; Kubota et al. 2015; Muir et al. 2014; Yadav et al. 2021). In particular, amphibians inhabiting high‐elevation environments are exposed to selective pressures associated with hypoxia, increased ultraviolet radiation, and low temperatures, for which they exhibit a wide range of morphological, physiological, and biochemical adaptations (Niu et al. 2024; Wang et al. 2018). These locally adapted traits may underlie a broad adaptive potential in montane species, potentially enhancing their resilience to climate change (Stöcklin et al. 2009).

The genotype‐environment association pattern identified in *M. bourgati* populations may underlie the species' adaptive capacity in response to future climate change. The projected changes in the extent and spatial arrangement of the three AUs within suitable areas provide valuable insight into this potential adaptive response. Under present‐day climatic conditions, AU1 and AU2 occupy comparable extents of suitable habitat (Figure 2b). However, future projections suggest that AU1 will be more severely impacted by the overall decline in habitat suitability under climate change scenarios. Under the most optimistic climate scenario (SSP1‐2.6)—the only one that does not predict the complete extinction of suitable habitat for the species—AU2 is predicted to persist across a broader extent of suitable habitat (Figure 5d). Considering the projected progressive reduction and disjunction in suitable areas culminating at the end of the century, AU1 is projected to primarily occupy the high‐elevation areas surrounding the Andringitra Massif, while the suitable habitat of AU2 is predicted to prevail in the mid‐elevation mountains near Ambohimahamasina, consistent with the NA elevational range where AU2 is currently found. This disjunction suggests that the currently heterogeneous climatic conditions of the Andringitra region occurring within a relatively restricted extent will become increasingly polarized under climate change. Future climate change is projected to amplify these contrasts leading to more extreme and spatially segregated environments, requiring the AUs to shift their ranges to track their respective suitable climatic niches. This pattern is further exemplified by the xeric‐adapted AU3, which already occupies an extremely restricted area at the present time and is projected to lose all suitable habitat within a few decades for the likely disappearance of drier environments from the region (Figure 5).

### 4.3 Maladaptation Risk and Conservation Implications

Given its microendemic distribution, characterized by a narrow range and likely ecological specialization, we assume that *M. bourgati* has a limited capacity for rapid adaptive responses to climate change. In fact, genetic offset calculations suggested that, even if *M. bourgati* succeeds in dispersing to climatically suitable areas in the coming decades, and despite exhibiting a diversified adaptive landscape across environmental gradients, the genetic changes required to maintain its current genotype‐environment relationships to future climates will be substantial, resulting in a high risk of maladaptation. Genetic offset values are projected to increase progressively towards the end of the century (Figure 2c). Considering the disjunction of suitable areas predicted by 2100 under the optimistic SSP1‐2.6 climate change scenario, genetic offset is projected to reach its maximum levels in the high‐elevation areas surrounding the Andringitra Massif (Figure 6c). In the mid‐elevation mountainous region of Ambohimahamasina, genetic offset is predicted to remain moderate to high, indicating that even persistence in these areas will require considerable genetic change from the species relative to its present‐day genotype‐environment relationships.

Overall, there is a significant risk that the pace of climate change may exceed both the dispersal potential and adaptive capacity of *M. bourgati* . Global climate change is occurring at an unprecedented rate, exposing species to velocities of environmental change that surpass anything they experienced throughout their evolutionary histories, therefore questioning their actual ability to either track or adapt to new suitable conditions (Bush et al. 2004; Foden et al. 2019; IPCC 2022; MacCracken 2009). At the same time, species are increasingly exposed to multiple pressures expected to act synergistically, such as habitat alteration and destruction, the expansion of invasive alien species, and emerging infectious diseases, further limiting their capacity to respond to new climatic conditions (Cohen et al. 2019; Di Febbraro et al. 2019; Jaureguiberry et al. 2022; Mondanaro et al. 2024).

Within this context, the PA network should enable species to respond to environmental alterations under conditions where pressures from anthropogenic activities are minimized. Concerning our study, although a larger proportion of climatically suitable areas for *M. bourgati* are projected to remain within the current boundaries of the PA network (Figure 7a,b), genetic offset values are predicted to be higher inside than outside these areas (Figure 7c). Notably, the mid‐elevation mountains near Ambohimahamasina—where *M. bourgati* is projected to retain most of its suitable habitat (Figure 6c)—fall almost entirely outside the current PA network. Although the species is projected to require substantial genetic changes to maintain its present‐day genotype‐environment relationship in these mountains in the future, this region is predicted to maintain the lowest risk of maladaptation compared to the rest of the species' potential future range. As such, this area may serve as a refugium, not only for this microendemic frog but also for other montane species currently inhabiting the Andringitra Massif. Although the dispersal corridor from the Andringitra Massif is currently protected by the Paysage Harmonieux Protégé du Corridor Forestier Ambositra–Vondrozo, the lack of formal protection of the Ambohimahamasina region could pose a significant risk to its long‐term survival.

The risk that climate change may generate mismatches between the current PA location and the future distribution of climatically suitable habitats and species is a well‐recognized conservation challenge (Hannah 2008; Thomas and Gillingham 2015). Recent estimates suggest that up to 40% of the world current terrestrial PAs may undergo shifts in present climatic zones by the end of the century (Cui et al. 2025). Recommendations for spatial conservation planning of PA networks in the face of climate change emphasize the protection of areas identified as climatic refugia and the maintenance of habitat connectivity to facilitate long‐distance species' dispersal (Ranius et al. 2023). Recent studies suggested that existing PAs in Madagascar are generally projected to experience lower climate change velocities compared to unprotected areas (Lai and Beierkuhnlein 2024), and that they will largely retain their effectiveness in conserving vertebrate biodiversity at least until 2070, assuming lack of limitations to species' dispersal (Coldrey and Turpie 2021). Further studies need to assess climate change effects on Madagascar's PAs and, although this generally optimistic scenario may apply to several species, it may not be valid across all biodiversity components (e.g., Bush et al. 2012; Vieilledent et al. 2013). It may also overlook species with narrow climatic niches or highly restricted distributions, such as microendemics, which hold high conservation values.

Traditionally, spatial conservation planning and assessments of species' vulnerability to climate change have relied on future projections of habitat suitability derived from ENMs to identify priority areas for conservation (Foden et al. 2019; Ranius et al. 2023). With the integrative approach proposed in this study, combining ENMs with GEA analyses, we emphasize the importance of considering multiple dimensions of biodiversity responses to climate change, for example explicitly including species' genetic adaptive capacity and the associated risk of maladaptation. In fact, our results highlighted a key limitation of grounding conservation strategies solely on habitat suitability, as even when species might succeed in tracking future climatic conditions, the genetic changes required to maintain their current genotype‐environment associations may be substantial. By integrating genomic information, our approach potentially enables the classification of climatically suitable areas according to their expected adaptive potential, thus allowing conservation efforts to prioritize locations where species are most likely to persist under future climate change.

### 4.4 Methodological Caveats and Perspectives

While our integrative approach provides a promising framework for predicting climate change effects on biodiversity, some limitations related to both the study system and the adopted analytical framework should be acknowledged, and the strategies adopted to mitigate them should be clearly outlined, as they also highlight opportunities to further improve this approach.

First, GEAs may be influenced by patterns of neutral genetic variation (Forester et al. 2018; Frichot et al. 2013). In this study, we characterized neutral genetic structure by testing for discrete clusters, IBD, and evidence of gene flow, and explicitly accounted for these factors in the GEA analyses. For example, population structure was incorporated in LFMM by specifying five latent factors, corresponding to the number of sampled populations, and a conservative outlier selection was applied by retaining only loci jointly detected by multiple GEA approaches (LFMM and RDA). In addition, the sensitivity of each method to outlier detection was assessed across a range of parameter settings.

Second, the limited spatial extent of the study area, relatively small sample size, and clustered sampling design reflect the extremely restricted distribution of this microendemic species and represent an intrinsic constraint of the study system, potentially reducing the statistical power of GEA approaches, although such methods are commonly applied to small sample sizes, particularly in nonmodel species (e.g., Chen et al. 2023; Ferreira et al. 2020). However, because we sampled virtually all localities where the species is confidently known to occur, our sampling likely captured the main geographic and environmental gradients occupied by the species. Moreover, the conservative analytical framework described above likely further mitigated some of these limitations, reducing the likelihood that the detected genotype‐environment associations are driven by neutral processes.

Finally, as is often the case for rare species, particularly in tropical systems, knowledge of the species' biology and natural history remains limited, especially with respect to dispersal capacity, which is central to our integrated approach. To account for this uncertainty, we implemented multiple dispersal rate scenarios within our modeling framework.

Accordingly, this study should be viewed as an example of the potential of this integrative approach, and our results should be interpreted as suggesting possible trends and hypotheses rather than providing definitive evidence. Future applications could strengthen this framework and increase its predictive value through broad geographic coverage and large sample sizes, the integration of more comprehensive genomic datasets, and the incorporation of direct information on dispersal and other relevant aspects of species' biology and natural history.

## Author Contributions

**Francesco Belluardo:** conceptualization (equal), formal analysis (equal), investigation (equal), methodology (equal), supervision (equal), validation (equal), visualization (equal), writing – original draft (equal), writing – review and editing (equal). **Mirko Di Febbraro:** conceptualization (equal), formal analysis (equal), funding acquisition (equal), methodology (equal), resources (equal), supervision (equal), validation (equal), writing – original draft (equal), writing – review and editing (equal). **Javier Lobón‐Rovira:** investigation (equal), writing – review and editing (equal). **Ivo Oliveira Alves:** investigation (equal), writing – review and editing (equal). **Malalatiana Rasoazanany:** investigation (equal), writing – review and editing (equal). **Franco Andreone:** investigation (equal), writing – review and editing (equal). **Gonçalo M. Rosa:** investigation (equal), writing – review and editing (equal). **Simone Giovacchini:** formal analysis (equal), validation (equal), writing – review and editing (equal). **Enrico Mirone:** formal analysis (equal), validation (equal), writing – review and editing (equal). **Pushpinder Singh Jamwal:** formal analysis (equal), validation (equal), writing – review and editing (equal). **Sandra Afonso:** supervision (equal), writing – review and editing (equal). **Michele Innangi:** formal analysis (equal), supervision (equal), validation (equal), writing – original draft (equal), writing – review and editing (equal). **Gabriella Sferra:** formal analysis (equal), supervision (equal), validation (equal), writing – review and editing (equal). **Emiliano Trucchi:** formal analysis (equal), supervision (equal), validation (equal), writing – review and editing (equal). **Alessandro Mondanaro:** formal analysis (equal), validation (equal), writing – review and editing (equal). **Francesco Carotenuto:** formal analysis (equal), validation (equal), writing – original draft (equal), writing – review and editing (equal). **Anna Loy:** formal analysis (equal), validation (equal), writing – review and editing (equal). **Angelica Crottini:** conceptualization (equal), funding acquisition (equal), methodology (equal), resources (equal), supervision (equal), writing – review and editing (equal).

## Funding

This work was supported by National Geographic Society (https://doi.org/10.13039/100006363), EC‐50656R‐18 to Francesco Belluardo and Fundação para a Ciência e a Tecnologia (https://doi.org/10.13039/501100001871), PTDC/BIA‐EVL/31254/2017 to Angelica Crottini.

## Conflicts of Interest

The authors declare no conflicts of interest.

## Supporting information




**Text S1:** Bioclimatic variables downscaling.
**Text S2:** Genomic data preparation, optimization of Genotype‐environment association analyses, and genetic offset calculation with Gradient Forest.
**Text S3:** Adaptive units clustering.

**Table S1:** Occurrence records and genetic samples analyzed in this study, with associated geographic coordinates and population assignments. See Figure 1 for geographic distribution of occurrence records, samples, and populations across the study area. ACP, Angelica Crottini's extraction codes.
**Table S2:** Root Mean Squared Error (RMSE) and *R* squared of the bioclimatic variables downscaling models' validation datasets.
**Table S3:** Climate variables used to calibrate ENMs along with data source.
**Table S4:** Optimization of de novo assembly parameters using the R80 approach, as described by Rivera‐Colón and Catchen (2022). Parameters ‐M and ‐n were set equal to each other and iteratively tested in the “denovo_map.pl” pipeline, ranging from values 1 to 12, using a subset of 12 samples. The optimal values (highlighted in bold) were selected based on the smallest positive change in the number of polymorphic loci present in at least 80% of samples (R80) between successive iterations.
**Table S5:** Summed values of the A1 and A2 validity indices (S) and their relative change compared to the previous *k* value between CLARA and hierarchical clustering algorithms.

## Data Availability

Raw sequence reads have been deposited to NCBI GenBank (BioProject: PRJNA1337429). Metadata files associated with sequencing, the SNPs datasets, and R scripts for IBD tests, ENM, and customized components of the Life on the Edge pipeline (Barratt et al. 2024) used for GEA analyses have been deposited to Dryad (https://doi.org/10.5061/dryad.cfxpnvxm5). Additional information is provided in the Supporting Information, including Tables S1–S5 (including the occurrence dataset), and detailed methodological descriptions and results (Text S1–S3).
