# Supplementary material for: Not Just a Matter of Space: Integrating Ecological Niche Modeling With Genotype‐Environment Associations Suggests High Maladaptation Risks Under Climate Change for a Microendemic Malagasy Frog
Source: Ecol Evol. 2026 May 24;16(5):e73664. doi: 10.1002/ece3.73664 (PMC13239544; doi:10.1002/ece3.73664)
Supplement: Supplementary file 1 — Text S1: Bioclimatic variables downscaling. Text S2: Genomic data preparation, optimization of Genotype‐environment association analyses, and genetic offset calculation with Gradient Forest. Text S3: Adaptive units clustering. [file ECE3-16-e73664-s001.docx]

**Supporting Information for:**

**Not just a matter of space: Integrating ecological niche modeling with genotype-environment associations suggests high maladaptation risks under climate change for a microendemic Malagasy frog.**

Francesco Belluardo, Mirko Di Febbraro, Javier Lobón-Rovira, Ivo Oliveira Alves, Malalatiana Rasoazanany, Franco Andreone, Gonçalo M. Rosa, Simone Giovacchini, Enrico Mirone, Pushpinder Singh Jamwal, Sandra Afonso, Michele Innangi, Gabriella Sferra, Emiliano Trucchi, Alessandro Mondanaro, Francesco Carotenuto, Anna Loy, Angelica Crottini

**Table of Contents:**

| **Text S1 - Bioclimatic variables downscaling** | Page 2 |
| --- | --- |
| **Text S2 - Genomic data preparation, optimization of**  **Genotype-environment association analyses, and genetic**  **offset calculation with Gradient Forest** | Page 7 |
| **Text S3 - Adaptive units clustering** | Page 13 |

**Text S1**

**Bioclimatic variables downscaling**

**1 Methods**

**1.1 The bioclimatic variables’ downscaling procedure**

The bioclimatic variables used in this study have an original spatial resolution of 1 x 1 km wide side for both the present and future scenarios. Unfortunately, this resolution was too coarse to represent the climatic preferences of the species. We decided therefore to statistically downscale the bioclimatic variables to a spatial resolution of 100 x 100 m. The procedure was performed by implementing a Machine Learning Algorithm (MLA) to find the mathematical relationship between any single bioclimatic variables, at their original resolution, and some other predictor variables’ maps at the same spatial resolution. The implemented model was then projected onto the predictor variables at the resolution of 100 x 100 m. To this aim, we used three kinds of different predictor variables’ maps: the topographic features of the study area at their native spatial resolution of 100 x 100 m; the NDVI maps at the spatial resolution of 300 x 300 m; and local wind data. In details, as regards the topography, we used the digital elevation model (DEM, Jarvis et al., 2008) of the study area and two other DEM-derived maps: the slope, i.e. the raster map of the territory’s incline, and the aspect, which represents the compass direction or azimuth of any single cell of the raster. These maps were computed by using the function terrain of the R package ‘terra’ version 1.7-71. For the NDVI maps, we used the Copernicus data (<https://land.copernicus.eu/en>) of all the 12 months in 2023, with a native resolution of 300 x 300 m and, then, downscaled them by using the cubic spline interpolation as implemented in the R package ‘terra’ (Hijmans, 2025). As regards the wind data, we considered both the mean wind speed and mean air density maps at 10, 50, 100, 150, 200 meters above sea level as provided by Global Wind Atlas version 4.0 (Davis et al., 2023). For the downscaling process, at first, we upscaled all the above-described predictor variables to the same spatial resolution of the bioclimatic variables (i.e. 1 x 1 km). Then, by overlapping cell by cell the bioclimatic with predictor variables, we created a numeric dataset in which, for any value of the climate we had corresponding values of the predictors. This dataset was used to train as many MLAs as the climate descriptors, where the response variable was, one at time, a specific bioclimatic variable and the predictors were the topographic features, the NDVI and the wind data. The computed models were, then, projected onto the maps of the predictor variables at the 100 m side wide cell resolution. In this way, we had the downscaled maps of the bioclimatic variables. We used this procedure to downscale both the present and the future climate and always used the same predictor variables. This means that for all the future scenarios too, we used the current topographic variables, the NDVI and the wind data for the year 2023. As regards the topographic data, we believe that in the next 80 years Madagascar will continue to be relatively stable as its topography did not change significantly since the Late Cretaceous (Emmel et al., 2012). As regards the use of current NDVI and wind data for predicting future scenarios, it was recently demonstrated the historical correlation between climate and vegetation growth (Fayech & Tarhouni, 2021; Zhe & Zhang, 2021) and climate and wind energy (Fan et al., 2021; Pryor & Barthelmie, 2010; Wu et al., 2020; Zeng et al., 2019). Hence, considering that the first future temporal interval is between 2011 and 2040 (please refer to the main manuscript), i.e. it includes the temporal interval of the predictor variables, it is reasonable there is still a functional correlation between current predictors and future climate. In any case, after training all the MLAs, we decided to discard any bioclimatic variables with at least one model showing a value of validation R^2^ lower than 0.9 among all the models including both current and future climate scenario.

As regards the current scenario, we statistically downscaled all the 19 bioclimatic variables. These downscaled maps were then used to perform correlation test between bioclimatic variables (see the main manuscript). After consolidating the number of useful variables to perform the Ecological Niche Model, for future scenarios, we used the following strategy. At first, we applied MLAs for downscaling the following variables: BIO 2, BIO 4, BIO 5, BIO 6, BIO 9, BIO 13, BIO 14 and BIO 15. Then, we decided not to use MLA for BIO 7 and BIO 3 but to compute them mathematically as the difference between BIO 5 and BIO 6, for BIO 7, and as the ratio between BIO 2 and BIO 7, for BIO 3. This allowed us to drastically reduce the computational time and hardware resource demand for all the considered climate models and Shared Socioeconomic Pathway scenarios (SSP).

The MLA we employed for modelling the relationship between the climate and the predictors was the Extreme Gradient Boosting (XGBoost, Chen & Guestrin, 2016). XGBoost efficiently combines multiple decision trees to yield a model with high prediction performance and reduced overfitting. This is done by a built-in supervised process minimizing the following objective function:

$$\begin{aligned} \mathrm{obj}\left( \emptyset\right)=\sum_{i} l\left( \hat{y}_{i} , y_{i} \right)\sum_{k} \Omega\left( f_{k} \right) \end{aligned}$$

The right side of the previous equation contains two components: the training loss (*l*), which measures the difference between the predicted ($\hat{y}_{i})$ and the observed values ($y_{i}$), i.e., the prediction error, and the regularization ($\Omega$), which represents the model’s complexity. The best model is the one including an ensemble of decision trees that minimizes both the prediction error and the model’s complexity by discarding uninformative trees. We performed the XGBoost parameters’ tuning by using a 5-fold cross validation strategy and chose the best parameters combinations by relying on the root mean squared error (RMSE) of the validation datasets. We trained and validated the XGBoost models by using the R package ‘caret’ (Kuhn, 2008).

**2 Results**

**2.1 Validation of the Machine Learning Algorithms**

For all the 19 bioclimatic variables of the present, we downscaled via XGBoost algorithm, and the validation R^2^ ranged from a minimum value of 0.9298 (BIO 4) and a maximum of 0.9978 (BIO 6). As regards the RMSE, the lowest value recorded was 0.0058 (BIO 3), whereas the highest value was 96.3222, represented by BIO 12 (**Table S2**). When considering the future climate scenarios, gathering all the temporal intervals, models and SSP, the lowest R^2^ value was 0.9183 for Bio 13, during the temporal interval 2071–2100, for the model GFDL-ESM4 with SSP 585, whereas the highest R^2^ was 0.9982 for BIO 6, during the time interval 2041–2070, with the model UKESM1-0-LL and SSP 126. The lowest RMSE was 0.1048 for BIO 6, during the time interval 2041–2070, with the model UKESM1-0-LL and SSP 126, whereas the highest was 20.4990 with BIO 13, during the time bin 2071–2100 and under the MRI-ESM2-0 model and SSP 585 scenario (**Table S2**). According to these results, none of the selected variables was excluded for all the following the analyses.

**3 References**

Chen, T., & Guestrin, C. (2016). XGBoost: A scalable tree boosting system. *Proceedings of the 22nd ACM SIGKDD International Conference on Knowledge Discovery and Data Mining*, *13-17-August-2016*, 785–794. https://doi.org/10.1145/2939672.2939785

Davis, N. N., Badger, J., Hahmann, A. N., Hansen, B. O., Mortensen, N. G., Kelly, M., Larsén, X. G., Olsen, B. T., Floors, R., Lizcano, G., Casso, P., Lacave, O., Bosch, A., Bauwens, I., Knight, O. J., Potter van Loon, A., Fox, R., Parvanyan, T., Krohn Hansen, S. B., … Drummond, R. (2023). The Global Wind Atlas: A high-resolution dataset of climatologies and associated web-based application. *Bulletin of the American Meteorological Society*, *104*(8), E1507–E1525. https://doi.org/10.1175/BAMS-D-21-0075.1

Emmel, B., Boger, S. D., Jacobs, J., & Daszinnies, M. C. (2012). Maturity of central Madagascar’s landscape — Low-temperature thermochronological constraints. *Gondwana Research*, *21*(2–3), 704–713. https://doi.org/10.1016/j.gr.2011.05.018

Fan, W., Liu, Y., Chappell, A., Dong, L., Xu, R., Ekström, M., Fu, T.-M., & Zeng, Z. (2021). Evaluation of global reanalysis land surface wind speed trends to support wind energy development using In situ observations. *Journal of Applied Meteorology and Climatology*, *60*(1), 33–50. https://doi.org/10.1175/JAMC-D-20-0037.1

Fayech, D., & Tarhouni, J. (2021). Climate variability and its effect on normalized difference vegetation index (NDVI) using remote sensing in semi-arid area. *Modeling Earth Systems and Environment*, *7*(3), 1667–1682. https://doi.org/10.1007/s40808-020-00896-6

Hijmans, R. J. (2025). *terra: Spatial Data Analysis*. https://CRAN.R-project.org/package=terra

Jarvis, A., Reuter, H. I., Nelson, A., & Guevara, E. (2008). *Hole-filled SRTM for the globe Version 4*. Available from the CGIAR-CSI SRTM 90m Database. http://srtm.csi.cgiar.org. Accessed on 01 February 2025.

Kuhn, M. (2008). Building predictive models in R using the caret package. *Journal of Statistical Software*, *28*(5). https://doi.org/10.18637/jss.v028.i05

Pryor, S. C., & Barthelmie, R. J. (2010). Climate change impacts on wind energy: A review. *Renewable and Sustainable Energy Reviews*, *14*(1), 430–437. https://doi.org/10.1016/j.rser.2009.07.028

Wu, J., Shi, Y., & Xu, Y. (2020). Evaluation and projection of surface wind speed over China based on CMIP6 GCMs. *Journal of Geophysical Research: Atmospheres*, *125*(22). https://doi.org/10.1029/2020JD033611

Zeng, Z., Ziegler, A. D., Searchinger, T., Yang, L., Chen, A., Ju, K., Piao, S., Li, L. Z. X., Ciais, P., Chen, D., Liu, J., Azorin-Molina, C., Chappell, A., Medvigy, D., & Wood, E. F. (2019). A reversal in global terrestrial stilling and its implications for wind energy production. *Nature Climate Change*, *9*(12), 979–985. https://doi.org/10.1038/s41558-019-0622-6

Zhe, M., & Zhang, X. (2021). Time-lag effects of NDVI responses to climate change in the Yamzhog Yumco Basin, South Tibet. *Ecological Indicators*, *124*, 107431. https://doi.org/10.1016/j.ecolind.2021.107431

*Disclaimer for the Global Wind Atlas version 4.0*

[Data/information/map] obtained from the Global Wind Atlas version 4.0, a free, web-based application developed, owned and operated by the Technical University of Denmark (DTU). The Global Wind Atlas version 4.0 is released in partnership with the World Bank Group, utilizing data provided by Vortex, using funding provided by the Energy Sector Management Assistance Program (ESMAP). For additional information: [https://globalwindatlas.info](https://globalwindatlas.info/)

**Text S2**

**Genomic data preparation, optimization of Genotype-environment association analyses, and genetic offset calculation with Gradient Forest**

**1 Methods**

**1.1 Genomic data preparation and sequencing**

Genomic DNA was extracted using a salt-extraction protocol modified from Enbody et al. (2021). DNA quantity and purity were assessed using a Qubit Fluorometer (Thermo Fisher Scientific, Waltham, MA, US) and an Epoch Microplate Spectrophotometer (BioTek Instruments, Winooski, VT, US), respectively. Samples yielding insufficient levels of DNA concentration and purity were re-extracted using the QIAamp DNA Micro kit (Qiagen, Venlo, NL) and re-evaluated using the same instruments. Double-digest Restriction-Site Associated DNA libraries were prepared at the Plateforme d’analyses génomiques of the Institut de Biologie Intégrative et des Systèmes (IBIS, Université Laval, Québec, CA), following the protocol described by Poland et al. (2012). Restriction digestion was performed using PstI and MspI restriction enzymes. A key modification to the original protocol involved the use of a blue Pippin (SAGE Science, Beverly, MA, US) to size libraries before PCR amplification (elution set between 50 and 65 min, on a 2% gel). Plate barcoding was applied to multiplex samples through inline barcodes at single-end reads, and paired-end sequencing (150 bp read length) was performed on a shared Illumina NovaSeq S4 lane at Génome Québec (Montréal, CA), following the approach described in Colston-Nepali et al. (2019).

**1.2 Genomic data assembly**

Stacks 2.6 (Catchen et al., 2013) was used to demultiplex and filter raw reads, perform de novo assembly, and SNPs calling. Specifically, demultiplexing, quality filtering, and adapter trimming were conducted using the ‘process_radtags’ module with default parameters for sliding window size and Phred quality score threshold. After demultiplexing, four samples from the Fivahona Velotsoa locality (ACP5302, ACP5307, ACP5309, and ACP5310) were excluded from the following analyses due to low number of retained reads (< 1 million; see **Fig. A1a**; Rivera-Colón & Catchen, 2022). The only remaining sample from this locality (ACP5273), which had slightly higher read numbers, was insufficient to represent the population on its own and was also excluded prior to de novo assembly (**Fig. A1a**).

De novo assembly parameters were optimized using a subset of 12 individuals selected from across all populations and with average sequencing depth relative to the entire dataset. Optimization followed the ‘R80’ method first described by Paris et al. (2017), which identifies parameter values that maximize the number of polymorphic loci in at least 80% of the samples. Following the procedure outlined by Rivera-Colón & Catchen (2022), we iterated the ‘denovo_map.pl’ pipeline across a range of values for the parameters -M and -n from 1 to 12, while keeping the two parameters equal to each other in each iteration. The optimal parameter values (-M = -n = 6) were identified as those corresponding to the lowest yet still positive increase in the number of R80 loci between successive iterations (**Table S4**; **Fig. A2**). De novo assembly and SNP calling on the full dataset were subsequently performed using the optimized parameters and the same Stacks pipeline. See the main manuscript for SNPs filtering parameters. To reduce the overall amount of missing data in individual genotypes, we followed the approach described by Cerca et al. (2021) and excluded three individuals prior to the final SNP filtering step. These individuals (ACP5288, ACP6817, and ACP6880) belonged to the largest population in the dataset (Andringitra Western Slopes; **Fig. 1**), exhibited low coverage levels, and were associated with coordinates where other individuals with lower missing data amounts were also available (**Table S1**; **Fig. A3**). We removed these samples because their absence was unlikely to compromise spatial representation and was expected to minimize potential biases in subsequent Genotype-environment association (GEA) analyses.

**1.3 Genotype-environment association analyses**

*1.3.1 LFMM parameter optimization and sensitivity*

To account for isolation-by-distance across the five analyzed populations of *M*. *bourgati*, we performed different LFMM analyses testing from 1 to 5 K latent factors and selected the optimal value based on the calibration of p-value distributions and genomic inflation factor values. To explore the sensitivity in outlier SNP detection relative to False Discovery Rate (FDR), we tested, for each environmental predictor, the effect of varying FDR thresholds (from 0.001 to 0.1) applied to q-values on the number of candidate SNPs. In addition, False Positive (FP) rates were estimated as the difference between the number of candidate SNPs at each FDR threshold and the number of SNPs detected at the most stringent threshold (FDR = 0.001), which was considered the set of true positives. Analyses were performed with the Life on the Edge (LotE) pipeline (Barratt et al., 2024).

*1.3.2 RDA optimization*

After performing RDA, we explored the method sensitivity in SNP selection by testing a range of standard deviation (SD) thresholds (from 1.0 to 3.5) used as selection cut-offs on RDA loadings. For each SD threshold, we extracted the number of candidate SNPs and estimated FP rates by comparing the number of SNPs detected at that threshold to the number of presumed true positives (i.e., selected at the most stringent SD threshold of 3.5). The analyses were performed with the LotE pipeline (Barratt et al., 2024).

**1.4 Genetic offset calculation with Gradient Forest**

To account for potential non-linear genotype-environment associations, we implemented a Gradient Forest (GF) approach as a complementary analysis to the RDA-based genetic offset calculation (**2.8 Genetic offset calculation** in the main manuscript). Allele frequencies were calculated for each of the 58 SNPs identified as candidates by GEA analyses across thirteen sampling units corresponding to unique coordinates of genotyped individuals (**Table S1**). Missing allele-frequency values were conservatively imputed using per-SNP mean frequencies across sampling units.

We fitted a GF model using the implementation function provided by Fitzpatrick et al. (2021) (<https://github.com/fitzLab-AL/geneticOffsetR>), with 1,000 trees and the same six climatic predictors used in the GEA analyses (**Table S3**). Genetic offset was then calculated as the Euclidean distance between current and future GF-transformed environmental scores on a per-pixel basis, following the same conceptual framework adopted for the RDA-based calculation (**2.8 Genetic offset calculation** in the main manuscript).

**2 Results**

**2.1 Genomic data assembly**

Excluding the samples from Fivahona Velotsoa (n = 5), removed after the ‘process_radtags’ filtering step (see **1.2 Genomic data assembly**), the remaining individuals retained an average of 8,321,944 reads (min = 1,146,526; max = 25,885,475; **Fig. A1a**). Among populations (excluding Fivahona Velotsoa), Andringitra Western Slopes (the population with the highest number of samples) showed the greatest variation in retained read counts, with median values ranging from 5,678,164 (Namoly) to 10,298,616 (Andringitra Eastern Slopes; **Fig. A1b**). See **Fig. A1c** and **d** for the percentages of input reads retained per individual and population.

Following de novo assembly and SNP calling on the full dataset using the ‘denovo_map.pl’ pipeline, a total of 1,008,510 loci were genotyped. The mean effective coverage per sample across the entire dataset was 28.7x (standard deviation = 12.2x, min =13.5x, max =56.7x). See **Fig. A3** for coverage distribution across samples and populations. After excluding three additional individuals from the Andringitra Western Slopes population before the final filtering with the ‘populations’ module (see **1.2 Genomic data assembly**; **Fig. A3**), the final dataset used for downstream analyses included 32 individuals and 6,381 SNPs.

**2.2 Sensitivity to outlier SNP detection**

Sensitivity analysis of LFMM revealed a consistent increase in the number of candidate SNPs with increasing FDR thresholds for all environmental predictors (**Fig. A4a**). Predictors Bio3, Bio4, and Bio13 consistently identified more candidate SNPs than the others across the range of thresholds, suggesting stronger association signals. Importantly, estimated FP rates remained low across all predictors and FDR values (**Fig. A4b**), indicating a limited FP risk even under less stringent thresholds.

For RDA, sensitivity analyses showed a constant increase in the number of outlier SNPs with decreasing values of SD thresholds across all environmental predictors (**Fig. A5d**), with Bio3, Bio4, and Bio13 again determining the highest numbers of candidate SNPs over the other predictors. FP rates remained relatively low throughout the tested range of SD thresholds (**Fig. A5c**). Both the number of candidate SNPs and the estimated FP rates exhibited abrupt peaks at specific SD values. These instabilities were effectively mitigated by selecting a conservative SD cutoff for the final analysis (see main manuscript for details).

**2.3 Genetic offset with Gradient Forest**

Overall temporal patterns of genetic offset inferred using the GF approach were broadly consistent with those obtained from the RDA-based analysis across all dispersal scenarios, showing a general increase from 2040 to 2100, although with some differences (**Fig. 2c**; **Fig. A6**). Under the SSP5-8.5 scenario, genetic offset values for AU1 and AU2 did not stabilize between 2070 and 2100, but instead continued to increase (**Fig. A6b**). In contrast, genetic offset values for AU3 were generally projected to be closer to those of the other AUs (**Fig. A6b**). This overall pattern was also recovered when incorporating a 200 m radius buffer around binary suitable patches (**Fig. A7**).

**3 References**

Barratt, C. D., Onstein, R. E., Pinsky, M. L., Steinfartz, S., Kühl, H. S., Forester, B. R., & Razgour, O. (2024). Life on the edge: A new toolbox for population‐level climate change vulnerability assessments. *Methods in Ecology and Evolution*, *15*(11), 2038–2058. https://doi.org/10.1111/2041-210X.14429

Catchen, J., Hohenlohe, P. A., Bassham, S., Amores, A., & Cresko, W. A. (2013). Stacks: An analysis tool set for population genomics. *Molecular Ecology*, *22*(11), 3124–3140. https://doi.org/10.1111/mec.12354

Cerca, J., Maurstad, M. F., Rochette, N. C., Rivera-Colón, A. G., Rayamajhi, N., Catchen, J. M., & Struck, T. H. (2021). Removing the bad apples: A simple bioinformatic method to improve loci-recovery in de novo RADseq data for non-model organisms. *Methods in Ecology and Evolution*, *12*(5), 805–817. https://doi.org/10.1111/2041-210X.13562

Colston-Nepali, L., Tigano, A., Boyle, B., & Friesen, V. (2019). Hybridization does not currently pose conservation concerns to murres in the Atlantic. *Conservation Genetics*, *20*(6), 1465–1470. https://doi.org/10.1007/s10592-019-01223-y

Enbody, E. D., Sprehn, C. G., Abzhanov, A., Bi, H., Dobreva, M. P., Osborne, O. G., Rubin, C. J., Grant, P. R., Grant, B. R., & Andersson, L. (2021). A multispecies BCO2 beak color polymorphism in the Darwin’s finch radiation. *Current Biology*, *31*(24), 5597–5604. https://doi.org/10.1016/j.cub.2021.09.085

Fitzpatrick, M. C., Chhatre, V. E., Soolanayakanahally, R. Y., & Keller, S. R. (2021). Experimental support for genomic prediction of climate maladaptation using the machine learning approach Gradient Forests. *Molecular Ecology Resources*, *21*(8), 2749–2765. https://doi.org/10.1111/1755-0998.13374

Paris, J. R., Stevens, J. R., & Catchen, J. M. (2017). Lost in parameter space: A road map for STACKS. *Methods in Ecology and Evolution*, *8*(10), 1360–1373. https://doi.org/10.1111/2041-210X.12775

Poland, J. A., Brown, P. J., Sorrells, M. E., & Jannink, J.-L. (2012). Development of high-density genetic maps for barley and wheat using a novel two-enzyme genotyping-by-sequencing approach. *PLoS ONE*, *7*(2), e32253. https://doi.org/10.1371/journal.pone.0032253

Rivera-Colón, A. G., & Catchen, J. (2022). Population genomics analysis with RAD, reprised: Stacks 2. In C. Verde & D. Giordano (Eds.), *Marine Genomics. Methods in Molecular Biology* (Vol. 2498, pp. 99–149). Humana. https://doi.org/10.1007/978-1-0716-2313-8_7

**Text S3**

**Adaptive units clustering**

**1 Clustering procedure used to delineate adaptive units (AUs)**

To delineate AUs from the multilocus adaptive signal, we clustered individuals in the space defined by Redundancy Analysis (RDA) scores of SNPs putatively under selection. On the retained RDA axes, we computed pairwise Gower dissimilarities with optimized variable weights (function ‘gawdis’ in the homonymous R package; w.type = ‘optimized’, 2,000 iterations), ensuring a balanced contribution of axes to the distance structure (de Bello et al., 2021). We then evaluated alternative clustering solutions for *k* ∈ [2–6] using two complementary algorithms applied to the dissimilarity matrix: (i) CLARA, a PAM-type partitional method suitable for large datasets, and (ii) agglomerative hierarchical clustering with Ward’s criterion (ward.D2). Both methods were implemented through ‘clusterbenchstats’ within the ‘fpc’ R package framework, with bootstrap-based stability assessment (bootmethod = ‘nselectboot’, 99 resamples; classification by centroid, medoid, and average distance; Hennig, 2019).

For each method–*k* combination, we computed the calibrated, aggregated cluster validity indexes A1 (within-cluster homogeneity) and A2 (between-cluster separation) following Akhanli & Hennig (2020). The number of clusters (*k*) was selected based on the largest relative improvement of the S index (i.e., A1 + A2) between consecutive *k* values. This elbow-like criterion jointly maximizes compactness and separation while avoiding an excessive number of clusters. The relative change was calculated as:

$$Relative change \left( \% \right)=\left\{ \begin{aligned} 100 if k=2 \\ 100\times\frac{S\left( k \right)-S\left( k-1 \right)}{\left| S\left( k-1 \right) \right|} \end{aligned} \right.if k \neq2$$

Accordingly, we selected *k* = 3 and CLARA clustering method as it offered a 1100% improvement in clustering validity metrics compared to *k* = 2 within the same closeting method (**Table S5**).

**2 References**

Akhanli, S. E., & Hennig, C. (2020). Comparing clusterings and numbers of clusters by aggregation of calibrated clustering validity indexes. *Statistics and Computing*, *30*(5), 1523–1544. https://doi.org/10.1007/s11222-020-09958-2

de Bello, F., Botta‐Dukát, Z., Lepš, J., & Fibich, P. (2021). Towards a more balanced combination of multiple traits when computing functional differences between species. *Methods in Ecology and Evolution*, *12*(3), 443–448. https://doi.org/10.1111/2041-210X.13537

Hennig, C. (2019). Cluster validation by measurement of clustering characteristics relevant to the user. In C. H. Skiadas & J. R. Bozeman (Eds.), *Data Analysis and Applications 1* (pp. 1–24). Wiley-ISTE. https://doi.org/10.1002/9781119597568.ch1
